# Single-cell analysis identifies a key role for *Hhip* in murine coronal suture development

**DOI:** 10.1038/s41467-021-27402-5

**Published:** 2021-12-08

**Authors:** Greg Holmes, Ana S. Gonzalez-Reiche, Madrikha Saturne, Susan M. Motch Perrine, Xianxiao Zhou, Ana C. Borges, Bhavana Shewale, Joan T. Richtsmeier, Bin Zhang, Harm van Bakel, Ethylin Wang Jabs

**Affiliations:** 1 Department of Genetics and Genomic Sciences, Icahn School of Medicine at Mount Sinai, New York, NY 10029 USA; 2 Icahn Institute for Data Science and Genomic Technology, Icahn School of Medicine at Mount Sinai, New York, NY 10029 USA; 3 Department of Anthropology, Pennsylvania State University, University Park, PA 16802 USA; 4 Mount Sinai Center for Transformative Disease Modeling, Icahn School of Medicine at Mount Sinai, New York, NY 10029 USA; 5 Department of Cell, Developmental and Regenerative Biology, Icahn School of Medicine at Mount Sinai, New York, NY 10029 USA; 6 Department of Pharmacological Sciences, Icahn School of Medicine at Mount Sinai, New York, NY 10029 USA; 7 Department of Pediatrics, Icahn School of Medicine at Mount Sinai, New York, NY 10029 USA; 8 Department of Genetic Medicine and Department of Pediatrics, Johns Hopkins School of Medicine, Baltimore, MD 21205 USA

**Keywords:** Bone development, Disease model, Embryology, Multicellular systems, RNA sequencing

## Abstract

Craniofacial development depends on formation and maintenance of sutures between bones of the skull. In sutures, growth occurs at osteogenic fronts along the edge of each bone, and suture mesenchyme separates adjacent bones. Here, we perform single-cell RNA-seq analysis of the embryonic, wild type murine coronal suture to define its population structure. Seven populations at E16.5 and nine at E18.5 comprise the suture mesenchyme, osteogenic cells, and associated populations. Expression of *Hhip*, an inhibitor of hedgehog signaling, marks a mesenchymal population distinct from those of other neurocranial sutures. Tracing of the neonatal *Hhip*-expressing population shows that descendant cells persist in the coronal suture and contribute to calvarial bone growth. In *Hhip*^*−/−*^ coronal sutures at E18.5, the osteogenic fronts are closely apposed and the suture mesenchyme is depleted with increased hedgehog signaling compared to those of the wild type. Collectively, these data demonstrate that *Hhip* is required for normal coronal suture development.

## Abstract (web summary)

Craniofacial development depends on formation and maintenance of sutures between bones of the skull. Here the authors identify enriched expression of the hedgehog inhibitor Hhip, specifically in the mesenchyme of the murine coronal suture, and show sutural dysgenesis in Hhip−/− mutants.

## Introduction

Sutures are critical sites of craniofacial growth and development. During suturogenesis, osteoprogenitors proliferate and differentiate directly from mesenchyme to osteoblasts within the osteogenic fronts (OFs) of adjacent skull bones in a process termed intramembranous ossification, which occurs in the absence of a cartilage template. The OFs are separated by suture mesenchyme (SM), which is maintained for most human sutures into adulthood when they eventually fuse after growth ceases. This process involves multiple interacting signaling pathways, including those for hedgehog (HH), fibroblast growth factor (FGF), ephrin (EPH/EFN), NOTCH, insulin-like growth factor (IGF), retinoic acid (RA), transforming growth factor/bone morphogenetic protein (TGF/BMP), and wingless-related integration site (WNT) signaling^1^. Misregulation of such pathways due to genetic or environmental insult can lead to suture dysgenesis, such as wider spacing or premature fusion between bones. Widening of sutures occurs in cleidocranial dysplasia caused by loss-of-function mutations in *RUNX2*, which encodes the master transcription factor for osteogenic differentiation^2,3^. In contrast, loss of SM between bones results in bony fusion or craniosynostosis and reduces the growth potential of that suture. Craniosynostosis is a significant source of human pathology, occurring in ~1 in 2500 births^4^. Genetic causes have been identified for ~25% of all cases and comprise more than 90 genes involved in a variety of signaling pathways and tissue developmental processes^5–7^. The coronal suture, between the frontal and parietal bones on each side of the skull, is fused in ~25% of craniosynostosis cases and is the suture most commonly affected in syndromic craniosynostosis cases^6,8^.

The coronal suture is a fascinating suture for study due to its unique biological features. It is the earliest calvarial suture to develop, separating the frontal and parietal bones as they grow from the supraorbital mesenchyme just above the eye^1^. In mammals it also lies at the boundary between the neural crest and mesoderm lineages, with the frontal bone derived from neural crest and the parietal bone and SM derived from mesoderm. Little or no mixing occurs between lineages along the length of the embryonic suture^9–11^. The mesoderm extends anteriorly over the neural crest for a short distance, and as the parietal and frontal bones expand, the parietal bone similarly extends to overlap the frontal bone with a narrow SM separating the bones. In contrast, in the frontal, sagittal, and lambdoid sutures the bones of the calvaria are arranged end-to-end and are separated by a wide SM at embryonic stages.

Various studies have compared RNA expression between human normal and synostotic sutures to identify genes with a role in suture dysgenesis^12^. However, these studies often rely on post-fusion tissues or bone-derived cells expanded in culture and may not reflect in vivo transcriptomes. Additionally, such studies cannot address how expression of these genes is organized in cell populations. To better understand suturogenesis at the transcriptional and cell population levels with the goal of identifying genes of developmental significance, we have previously applied single-cell and bulk RNA-seq analyses to the murine frontal suture at embryonic day (E)16.5 and E18.5^13^.

In the current study, we apply these methods to the murine coronal suture. We identify major cell populations comprising SM and OFs of the coronal suture, including populations that differ from those of the frontal suture at the same ages^13^. We find that expression of the gene encoding hedgehog interacting protein (*Hhip*) is highest in a coronal SM population between E16.5 and E18.5. This is specific to the coronal suture because in contrast, *Hhip* expression is highest in the OFs in the other neurocranial sutures (frontal, sagittal, and lambdoid). *Hhip* encodes an inhibitor of HH ligands and is induced by HH signaling as a component of negative feedback loops regulating the pathway^14^. We perform tracing of the neonatal *Hhip*-expressing population showing that descendant cells persist in the wild type (WT) coronal SM and eventually contribute to calvarial bone growth. In *Hhip*^*−/−*^ mutant mice, delays in chondrocyte maturation and subsequent ossification of digits, sternum, and vertebrae, which develop from cartilaginous templates in a process termed endochondral ossification, have been described (see Supplementary Experimental Data in reference 14)^14^, but defects in intramembranous ossification have not been reported. We examine the coronal suture in *Hhip*^*−/−*^ mutants and uncover a phenotype of SM depletion. These findings provide key insights into the role of HH signaling in coronal suturogenesis.

## Results

### Single-cell RNA-seq analysis delineates the wild type coronal suture population structure

The coronal suture assumes its definitive morphology of overlapping parietal and frontal bones in the mouse during late embryonic development, E16.5–E18.5. At E16.5 the overlap of frontal and parietal bones is at an early phase and is fully established by E18.5, the final day of murine embryogenesis in C57BL/6 J, the strain used in this study. To identify the cell populations present during this transitional period we analyzed WT coronal suture development by single-cell RNA-seq (scRNA-seq) analysis of four libraries, consisting of two replicates at E16.5 and E18.5. Libraries were derived from strips of coronal sutures spanning the overlapping frontal and parietal bones, including the OFs of each bone and the intervening SM (Supplementary Fig. 1a). Extrasutural tissues were removed to the extent possible to enrich for sutural populations.

Analyzing all four libraries combined, we identified 14 cell populations (Supplementary Fig. 1b, d) by reference to published cell type atlases^15–19^ and our previously published study of the murine frontal suture^13^. These populations were broadly consistent with recent analyses of the murine coronal suture at E15.5 and E17.5^20^ and included SM and osteoblast populations as well as hematopoietic lineages, vascular cells, and chondrocytes. The majority of cells were part of a supercluster that included suture-specific populations (i.e., SM, osteoblasts; denoted as “CS” for “coronal suture” in Supplementary Fig. 1b) and comprised 56% and 86% of cells at E16.5 and E18.5, respectively (Supplementary Fig. 1b, c). Hematopoietic, vascular, and chondrocyte populations were common to both ages. Chondrocytes presumably were derived from the parietal cartilage (tectum transversum) present at the base of the coronal suture^21^.

### Definition and mapping of cell populations at E16.5

**Fig. 1:**
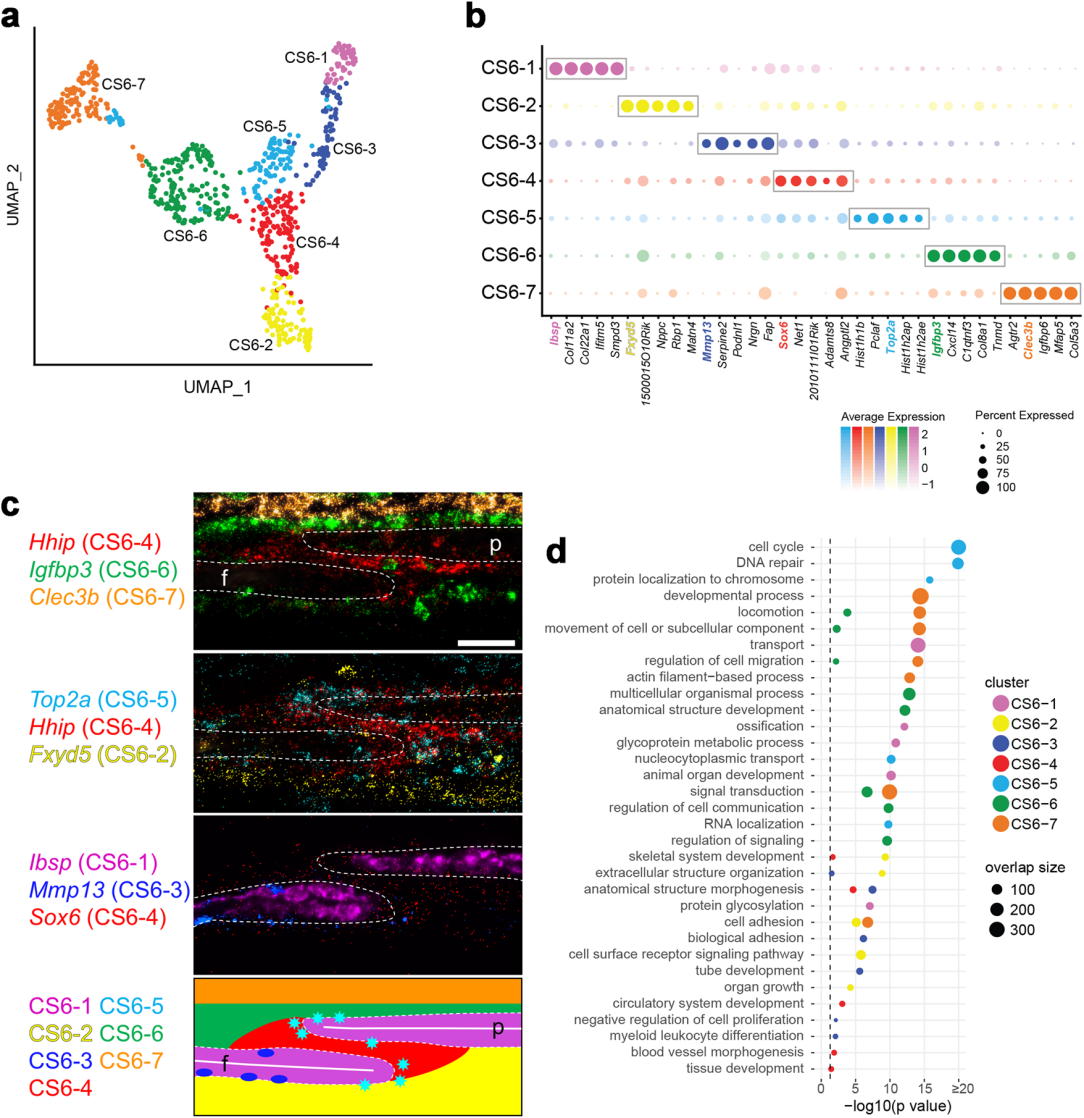
scRNA-seq analysis of the wild-type coronal suture at E16.5. **a** Uniform Manifold Approximation and Projection (UMAP) plot of cell clusters detected by unsupervised graph clustering of suture-specific populations (i.e., suture mesenchyme, osteoblasts) from two replicates at E16.5. **b** Average expression of the top five differentially expressed marker genes (boxed) ranked by fold change (FC) (FDR % 0.01, lnFC R 0.1) for suture-specific populations. Genes selected for smFISH in **c** are colored according to their population membership. **c** Localization of populations by smFISH. Each pseudo-colored panel shows an individual section hybridized with probes for the indicated genes. The schematic summarizes the spatial distribution of populations, color-coded as in smFISH. Dashed outlines indicate frontal (f) and parietal (p) bones; white horizontal lines indicate osteoid. Sections are in the transverse plane. smFISH was performed on three independent samples with similar results. Scale bar, 50 μm. **d** Significant GO BP categories of population-specific expression signatures. Gene Ontology enrichment was performed with ranked query and multiple testing analytical correction (*p* ≤ 0.05, right of dashed line).

To further resolve the composition of the supercluster we performed additional UMAP analyses of the associated cells at each development stage. At E16.5 the suture-specific supercluster consisted of seven distinct populations expressing known markers for two potential mesenchyme populations, osteoblasts, hypodermis, and dura mater^13,16–19^ (Fig. 1a). To determine the spatial relationships of these populations we used multiplexed single-molecule fluorescence in situ hybridization (smFISH) for genes within the top ten significant marker genes of each population (Fig. 1b, Supplementary Fig. 2d, and Supplementary Dataset 1). Marker genes were defined by their relative specificity to a given population compared to all other populations. Additionally, we chose more highly expressed marker genes for mapping to facilitate detection. Two populations comprised the mesenchyme in the suture, CS6-4 (coronal suture E16.5 population 4) and CS6-6. CS6-4 consisted of SM between the frontal and parietal bones and extending beyond the OFs and was enriched for *Sox6* and *Erg* expression (Fig. 1c and Supplementary Fig. 2a). This population also expressed *Hhip* as a lower-ranked marker gene. However, *Hhip* was a highly ranked marker gene for a similar SM population at E18.5, and so its expression also was mapped at E16.5 to provide a point of comparison between the two ages. CS6-6 consisted of an ectocranial, or outer, layer of mesenchyme over the frontal and parietal bones and was enriched for *Igfbp3* expression.

Three populations comprised the OFs and osteoblasts. The first consisted of proliferating cells at the leading edge of the frontal and parietal bones and was enriched for *Top2a* expression (CS6-5; Fig. 1c and Supplementary Fig. 2a). The second consisted of the major population of the frontal and parietal bones and was enriched for *Ibsp* expression, an early osteoblast marker (CS6-1). The third consisted of a smaller population of osteoblasts principally found on the endocranial or inner surface of the frontal bone, enriched for *Mmp13* expression (CS6-3). This was not a frontal bone-specific suture population, as *Mmp13* also was expressed away from the suture in more mature regions of frontal bones, where high *Mmp13* and *Ibsp* expression were inversely correlated on trabecular bone (Supplementary Fig. 2b). Also, in regions of more mature parietal bone distant from the suture, *Mmp13* was expressed on the endocranial surface, while *Ibsp* was expressed on the ectocranial surface (Supplementary Fig. 2b). Two other populations were external to the suture. The hypodermis was superficial to the ectocranial mesenchyme and was enriched for *Clec3b* expression (CS6-7). The dura mater was endocranial to the suture and was enriched for *Fxyd5* expression (CS6-2). E16.5 single-cell populations are summarized in Supplementary Table 1.

To characterize the transcriptional programs particular to individual populations we performed Gene Ontology (GO) analysis of the marker genes distinguishing each population (Fig. 1d and Supplementary Dataset 1). The main SM population (CS6-4) was enriched for anatomical structure morphogenesis and skeletal system and tissue development. The ectocranial mesenchyme (CS6-6) was enriched for regulation of cell communication, signal transduction, and positive regulation of insulin-like growth factor receptor signaling pathway. The proliferating OF population (CS6-5) was enriched for cell cycle and related processes. The major osteoblast population (CS6-1) was enriched for ossification, collagen fibril organization, protein glycosylation, and protein hydroxylation. The minor osteoblast population expressing *Mmp13* (CS6-3) was enriched for anatomical structure morphogenesis, biological adhesion, and collagen catabolism.

### Definition and mapping of cell populations at E18.5

**Fig. 2:**
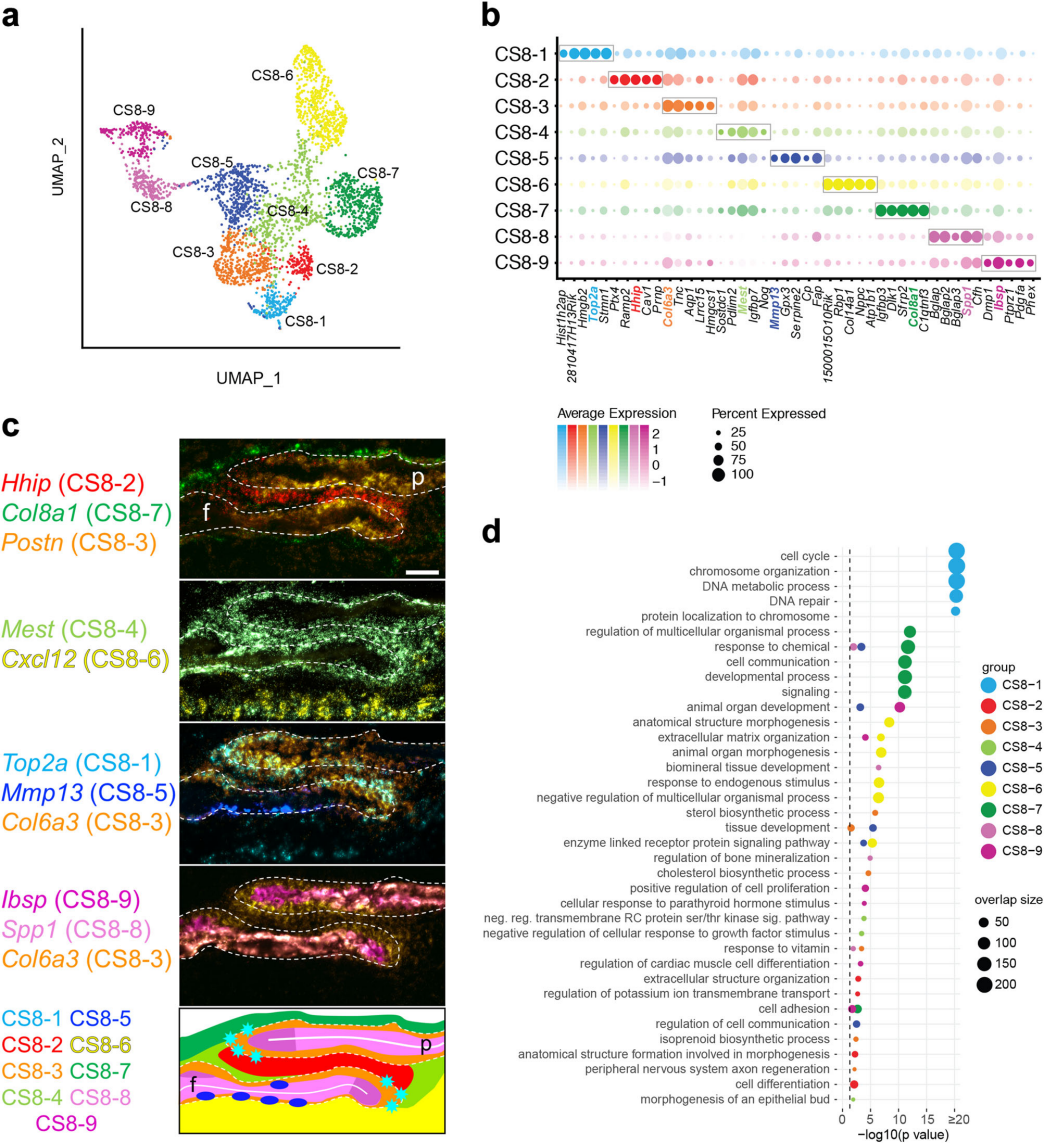
scRNA-seq analysis of the wild-type coronal suture at E18.5. **a** UMAP plot of cell clusters detected by unsupervised graph clustering of suture-specific populations (i.e., suture mesenchyme, osteoblasts) from two replicates at E18.5. **b** Average expression of the top five differentially expressed marker genes (boxed) ranked by FC (FDR % 0.01, lnFC R 0.1) for suture-specific populations. Genes selected for smFISH in **c** are colored according to their population membership. For CS8-6, *Cxcl12*, which is within the top ten markers of this population, was used for its known expression in dura mater^13^. **c** Localization of populations by smFISH. Each pseudo-colored panel shows an individual section hybridized with probes for the indicated genes. The schematic summarizes the spatial distribution of populations, color-coded as in smFISH. Dashed outlines indicate frontal (f) and parietal (p) bones; white horizontal lines indicate osteoid. Sections are in the transverse plane. smFISH was performed on three independent samples with similar results. Scale bar, 50 μm. **d** Significant GO BP categories of population-specific expression signatures. Gene Ontology enrichment was performed with ranked query and multiple testing analytical correction (*p* ≤ 0.05, right of dashed line).

At E18.5 the suture-specific supercluster consisted of nine distinct populations (Fig. 2a). We determined the spatial relationships of these populations using smFISH for significant marker genes (Fig. 2b, Supplementary Fig. 3b, and Supplementary Dataset 1). Three populations comprised the mesenchyme within the suture. One consisted of the mesenchyme between the overlapping frontal and parietal bones and was enriched for *Hhip* and *Cd34* expression (CS8-2; Fig. 2c and Supplementary Fig. 3a). The second consisted of mesenchyme adjacent to the OFs and the region of overlap between the bones and was enriched for *Mest* expression (CS8-4) that also overlapped the *Hhip* expression domain. The third consisted of an ectocranial layer of mesenchyme over the frontal and parietal bones and was enriched for *Col8a1* expression (CS8-7).

Five populations comprised the OFs and differentiated osteoblasts. The first consisted of proliferating cells at the leading edge of the frontal and parietal bones and was enriched for *Top2a* expression (CS8-1; Fig. 2c and Supplementary Fig. 3a). The second was located along the periosteal surfaces, particularly within the region of frontal and parietal overlap and was enriched for *Col6a3* and *Postn* expression (CS8-3). Expression of *Col6a3* appeared stronger than *Postn* towards the OFs. This population also was enriched for expression of *Fgfr2*, which is known to be expressed in OFs and periosteal surfaces of frontal and parietal bones^22–24^ (Supplementary Dataset 1). The third consisted of osteoblasts extending from the OFs along the osteoid of the frontal and parietal bones and was enriched for *Ibsp* expression (CS8-9). The fourth extended away from the suture along the bone distal to *Ibsp*-expressing osteoblasts and was enriched for expression of *Spp1*, a marker of more mature osteoblasts (CS8-8). The fifth principally extended along the endocranial surface of the frontal bone and was enriched for *Mmp13* expression (CS8-5). The final population comprised the dura mater endocranial to the suture and was enriched for *Cxcl12* expression (CS8-6). E18.5 single-cell populations are summarized in Supplementary Table 1.

We further examined the relationship of the proliferating CS8-1 population to the adjacent CS8-2 and CS8-3 and to other populations. While all other populations contained cells expressing proliferation markers such as *Mki67* at a low frequency, CS8-1 was primarily distinguished by the widespread expression of genes related to proliferation (Fig. 2b and Supplementary Fig. 4a). CS8-1 shared expression of a subset of genes with CS8-2 and CS8-3 that distinguished them from the remaining suture populations, suggesting that it may represent a transitional population between the SM population CS8-2 and the periosteal population CS8-3 (Supplementary Fig. 4b). CS8-1 also expressed genes not related to proliferation that distinguished it from both CS8-2 and CS8-3, further indicating that it is a distinct population.

We performed GO analysis of the marker genes distinguishing each population identified through differential expression analysis (Fig. 2d and Supplementary Dataset 1). For the SM populations, the *Hhip*-expressing SM (CS8-2) was enriched for extracellular structure organization, cell adhesion, cell differentiation, and skeletal system development. The *Mest*-expressing SM (CS8-4) was enriched for negative regulation of transmembrane receptor protein serine/threonine kinase signaling pathway and negative regulation of cellular response to growth factor stimulus. The ectocranial mesenchyme (CS8-7) was enriched for cell communication, cell signaling, and cell migration.

For the OF and osteoblast populations, the GO terms confirmed their diverse roles in skeletal development. The proliferating population (CS8-1) was enriched for cell cycle and related processes. The periosteal population (CS8-3) was enriched for sterol, cholesterol, and isoprenoid biosynthetic processes. The *Ibsp*-expressing osteoblast population (CS8-9) was enriched for extracellular matrix organization and positive regulation of cell proliferation. The *Spp1*-expressing osteoblast population (CS8-8) was enriched for biomineral tissue development. The *Mmp13*-expressing osteoblast population (CS8-5) was enriched for tissue development and collagen metabolic process.

### Population complexity in the coronal suture increases between E16.5 and E18.5

We matched the cell populations at E16.5 with those at E18.5 based on significant expression signature overlaps (Supplementary Fig. 3c and Supplementary Table 1). Most SM and osteoblast-related populations were clearly similar between E16.5 and E18.5, including the major *Hhip*-expressing SM populations (CS6-4 and CS8-2), ectocranial SM populations (CS6-6 and CS8-7), *Top2a*-expressing proliferating populations (CS6-5 and CS8-1), *Mmp13*-expressing osteoblasts (CS6-3 and CS8-5), and the dura mater populations (CS6-2 and CS8-6). *Ibsp*-expressing osteoblasts at E16.5 (CS6-1) matched with both *Ibsp*-expressing osteoblasts (CS8-9) and more differentiated *Spp1*-expressing osteoblasts (CS8-8) at E18.5. We did not identify discrete populations of *Mest*-expressing SM cells (CS8-4) or *Col6a3*-expressing periosteal cells (CS8-3) at E16.5, but *Mest* and *Col6a3* are expressed at E16.5 in similar domains as at E18.5 (Supplementary Fig. 2c), and were marker genes for CS6-4 and CS6-3, respectively. These differences may reflect changes in the numbers of cells in each of these populations or a lack of sufficient specificity of marker gene expression at this age. *Clec3b*-expressing hypodermis (CS6-7) was not identified at E18.5. This was likely due to easier and more effective removal of extrasutural tissue such as the hypodermis at E18.5 compared to E16.5.

### Ectocranial, periosteal, and osteoblast populations are enriched for craniosynostosis genes

Approximately 57 genes have been identified that cause coronal craniosynostosis when mutated in humans^5,7^ (Supplementary Dataset 2). We determined the degree of enrichment of these genes among the cell populations at each age. At E16.5 significant enrichments were found in the ectocranial mesenchyme (CS6-6; *Cyp26b1*, *Igf1r*, *Lmx1b*, *Stat3*, *Twist1*, *Zic1*; *P* = 0.013) and the *Mmp13*-expressing osteoblasts (CS6-3; *Alpl*, *Atr*, *Fgfr2*, *Fgfr3*, *Kat6a*, *Kmt2d*; *P* = 0.006). UMAPs highlighting the expression patterns for each of these genes are shown in Supplementary Fig. 5a. At E18.5 significant enrichments were found in the ectocranial mesenchyme (CS8-7; *Abcc9*, *Cyp26b1*, *Fbn1*, *Gpc3*, *Igf1r*, *Stat3*, *Twist1*, *Zic1*; *P* = 0.007), the *Mmp13*-expressing osteoblasts (CS8-5; *Alpl*, *Fgfr3*; *P* = 0.044), and the periosteal population (CS8-3; *Fgfr2*, *Flna*, *P4hb*; *P* = 0.029). If we used 96 genes currently associated with craniosynostosis of any suture^5,7^, these five populations also were enriched significantly. In addition, the *Ibsp*-expressing osteoblast population at E18.5 was enriched significantly (CS8-9; *B3gat3*, *Bmp2*, *Fam20c*, *Ihh*, *Irx5*, *Phex*, *Sec24d*, *Sh3pxd2b*; *P* = 0.001). UMAPs highlighting the expression patterns for each of these human craniosynostosis genes are shown in Supplementary Fig. 5b.

We repeated the enrichment analysis for 23 genes that cause coronal craniosynostosis when mutated in mice, of which 11 are models of human disease^25,26^ (Supplementary Dataset 2). At E16.5 significant enrichments were found in the ectocranial mesenchyme (CS6-6; *Axin2, Fgf9, Lmx1b, Twist1*; *P* = 0.001) and the *Mmp13*-expressing osteoblasts (CS6-3; *Alpl, Fgfr2, Fgfr3; P* = 0.005*)*. At E18.5 significant enrichments were found in the *Mmp13*-expressing osteoblasts (CS8-5; *Alpl*, *Fgfr3, Runx2*; *P* = 0.0002). If we used 31 genes associated with craniosynostosis of any suture in mice^25,26^, only the *Mmp13*-expressing osteoblast populations were enriched significantly. These two populations were common to the human analysis, but our finding of fewer populations enriched for murine craniosynostosis genes compared to human may be due to the lower number of mouse genes available for analysis. Overall, these enrichment profiles suggest that genes causing craniosynostosis function in different sutural cell populations and thus through varied mechanisms.

### *Hhip* expression distinguishes the coronal suture mesenchyme from other calvarial sutures

**Fig. 3:**
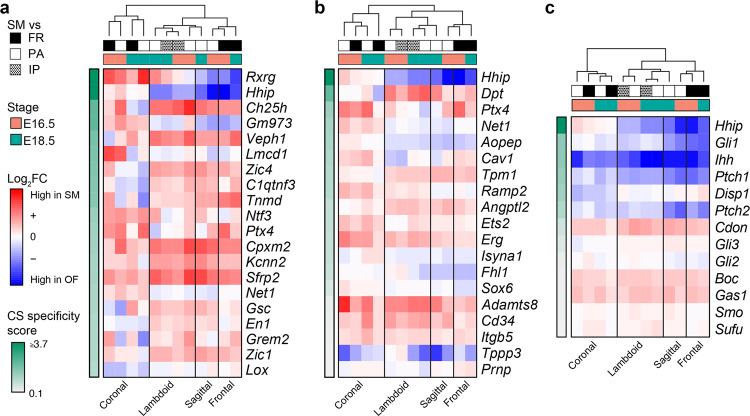
Enrichment of *Hhip* expression in SM is specific to the coronal suture. **a** Average bulk gene expression changes of the significant marker genes (rows) of all SM populations across the four calvarial sutures. Expression changes are shown as the log2FC between the SM and osteogenic fronts (OF) at E16.5 and E18.5, ranked by their ‘coronal specificity score’ (CS), representing the difference between the average expression across the coronal suture data and the average expression across the remaining sutures (lambdoid, sagittal, and frontal). Frontal OF (FR), parietal OF (PA), interparietal OF (IP). **b** Average bulk gene expression changes of the combined top ten marker genes of SM populations CS6-4 and CS8-2 across the four calvarial sutures. Expression changes are shown as in **a**. **c** Average bulk gene expression changes of key hedgehog pathway genes across the four calvarial sutures. Expression changes are shown as in **a**.

Because the molecular processes and cellular composition of SM are poorly characterized compared to those of osteoblast differentiation, we focused on identifying unique features of the coronal SM populations. The coronal suture is morphologically distinct from the other calvarial sutures in that the bones overlap during the embryonic period studied, which may be reflected by a distinct transcriptional signature. We intersected the significant marker genes from all single-cell, mesenchymal populations (CS6-4, CS6-6, CS8-2, CS8-4, and CS8-7; Supplementary Dataset 1) with our bulk RNA-seq datasets of the calvarial sutures (coronal, frontal, sagittal, and lambdoid sutures)^27,28^. These bulk datasets contain SM and OF gene expression profiles of each suture to allow identification of differential gene expression between SM and OFs. This intersection of single-cell and bulk RNA-seq data ranked single-cell, mesenchymal marker genes by the degree to which their average expression was enriched in the coronal SM compared to other sutures (Fig. 3a). *Hhip*, a marker of CS6-4 and CS8-2, was unique in that it was enriched only in the SM of the coronal suture whereas it was enriched in the OFs of the other calvarial sutures (Fig. 3a). In contrast, the other mesenchymal genes typically were enriched in the SM of all or most calvarial sutures, and therefore were not transcriptionally distinct markers of coronal SM. We repeated this analysis with an aggregate list of the combined top ten marker genes of the major SM populations, CS6-4 and CS8-2. *Hhip* was the gene most significantly enriched in coronal SM (Fig. 3b). In contrast, other genes such as *Angptl2*, *Adamts8*, *Cd34*, *Erg*, *Ets2*, and *Itgb5* were enriched in the SM of all calvarial sutures (Fig. 3b). *Hhip* is a component of the HH pathway. To explore this pathway further we made a similar comparison between a list of key members of the HH pathway and bulk SM and OF gene expression in all calvarial sutures. Expression of the Indian hedgehog (*Ihh*) ligand, its receptors *Ptch1* and *Ptch2*, and the HH transcriptional target *Gli1*, was enriched in the osteogenic fronts of all calvarial sutures (Fig. 3c). In agreement with this finding, *Ihh* was enriched in single-cell osteoblast populations CS6-1 and CS8-9 (Supplementary Dataset 1).

### The *Hhip*-expressing population persists postnatally and contributes to calvarial bone growth

**Fig. 4:**
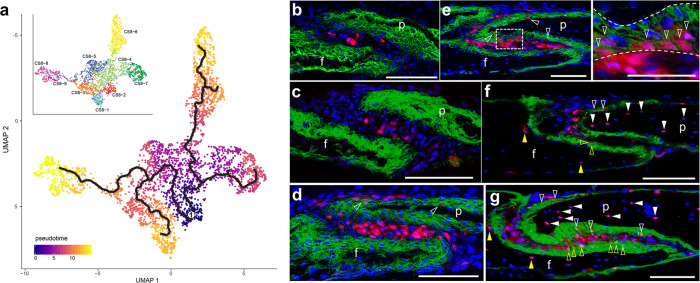
Fate mapping of *Hhip*-CreERT2-expressing suture mesenchyme. **a** Graph abstraction of the relationships between CS8-1 to CS8-9. Inset shows coloring by cluster identity. The root state of the inferred trajectory is indicated by the number 1 at the center of the cells with the earliest pseudotime. **b** TdTomato-expressing (tdTomato + ) cells at postnatal day (P)2 after 1 day of induction. *n* = 3. **c** TdTomato+ cells at P3 after two consecutive days of induction. *n* = 3. **d** TdTomato+ cells at P6 after five consecutive days of induction. *n* = 1. **e** TdTomato+ cells at P30 after 1 day of induction at P1. Dashed box is enlarged at right to show cluster of tdTomato+ osteoblasts within ALPL domain (dashed lines). *n* = 1. **f** TdTomato+ cells at P90 after two consecutive days of induction at P1 and P2. *n* = 3. **g** TdTomato+ cells at P90 after five consecutive days of induction between P1-P5. *n* = 2. White and yellow empty/solid arrowheads indicate tdTomato+ osteoblasts/osteocytes in parietal and frontal bones, respectively. f frontal bone, p parietal bone. Sections are in the sagittal plane. Scale bars, 100 μm except inset in **e**, 50 μm.

We explored the potential relationships between the *Hhip*-expressing and other coronal suture single-cell populations. We first identified potential ligand/receptor interactions between populations by their gene expression using CellPhoneDB^29^ at E18.5, when more cell populations were defined (Supplementary Fig. 6). *Hhip*-expressing CS8-2 had the most potential interactions with dura mater (CS8-6), ectocranial mesenchyme (CS8-7), and early osteoblasts (CS8-9), but CS8-2 showed fewer interactions overall compared to other populations (Supplementary Fig. 6a). Dura mater (CS8-6) and ectocranial mesenchyme (CS8-7) had the largest number of potential mutual interactions, but are also the most widely separated populations. However, both had similar numbers of potential interactions with the mesenchymal (CS8-4) and osteoblast (CS8-9, CS8-8) populations. We then focused on potential ligand/receptor interactions specifically involving genes expressed in CS8-2 (Supplementary Fig. 6b). Prominent potential interactions, particularly with populations nearest to CS8-2, included a variety of collagen/integrin complexes and members of the FGF and TGF signaling pathways. With regards to HH signaling, IHH and HHIP showed a notable interaction. We then inferred an approximate graph abstraction at E18.5 (Fig. 4a). The trajectory graph from CS8-2 consisted of two main branches. One branch extended through CS8-5 and connected to proliferating CS8-1, periosteal CS8-3, and osteoblastic CS8-8 and CS8-9. The other branch extended through CS8-4 before bifurcating into ectocranial mesenchyme CS8-7 and dura mater CS8-6. Both these analyses suggest complex interactions between coronal suture populations during development.

To elucidate the connection between the *Hhip*-expressing and other coronal suture populations in vivo we crossed *Hhip*-CreERT2 mice with the Ai14 reporter line^30^ and induced recombination in early postnatal pups by tamoxifen induction. Daily tamoxifen induction starting at P1 for one, two, or  five days showed that *Hhip*-CreERT2 activity was dose responsive and highly specific to the SM (Fig. 4b–d). Furthermore, after five days of induction there was negligible labeling of alkaline phosphatase (ALPL)-expressing preosteoblasts and osteoblasts despite widespread labeling of adjacent SM that extended around the parietal OF into ectocranial mesenchyme. These findings suggested that labeled SM cells did not differentiate rapidly to osteoblasts during the early postnatal period (Fig. 4d). Thirty days after induction at P1, the frequency of labeled cells in the SM was increased greatly but relatively few cells had differentiated to osteoblasts (Fig. 4e). Ninety days after induction at P1 and P2, labeled cells were present still in the SM but now were incorporated as osteoblasts and osteocytes in the frontal and parietal bones (Fig. 4f). Similarly, 90 days after daily induction between P1 and P5, labeled cells were widespread in the SM, osteoblasts, and osteocytes (Fig. 4g).

### *Hhip* is required for normal coronal suture development

**Fig. 5:**
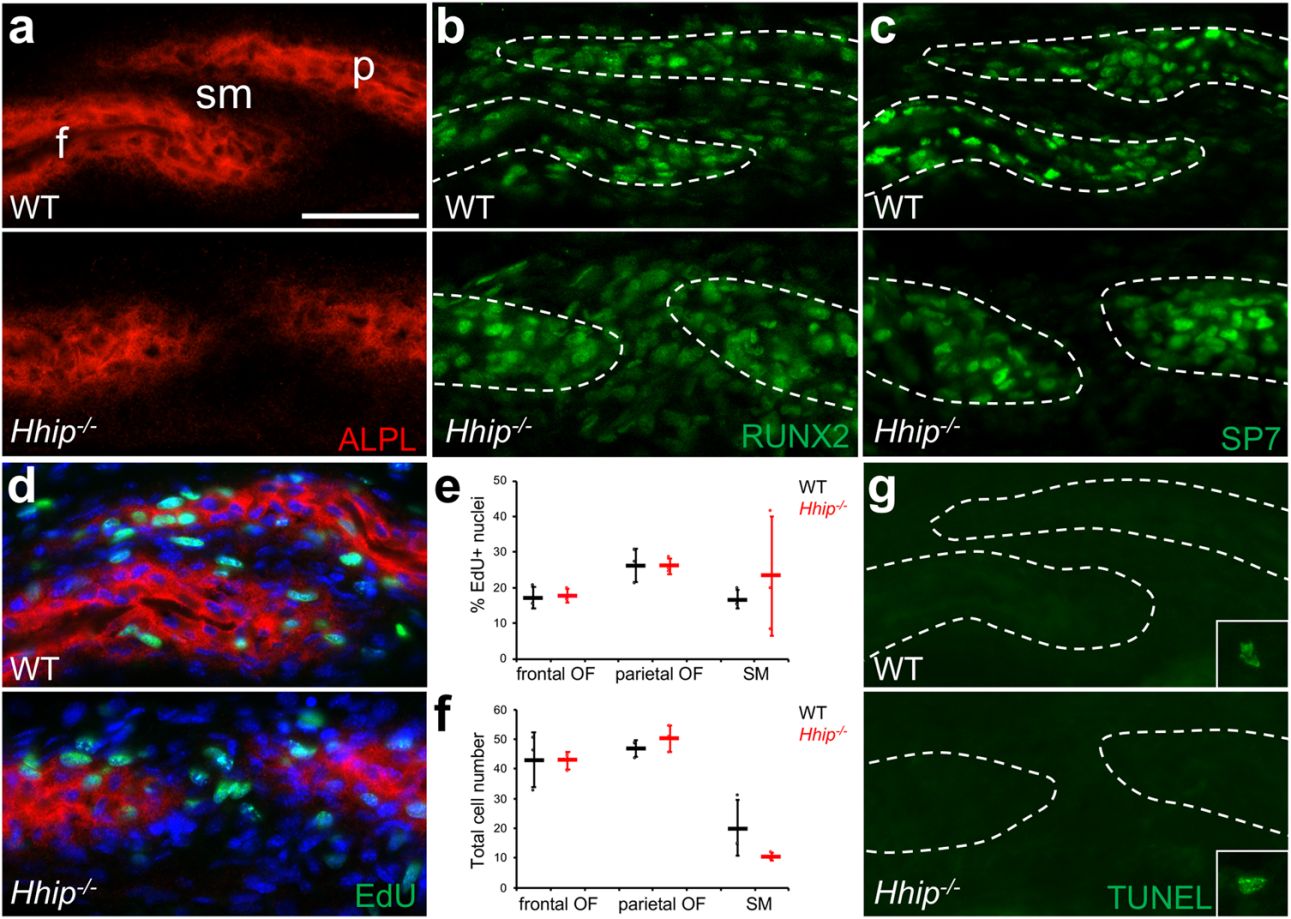
Coronal suture dysgenesis in *Hhip*^*−/−*^ mice at E16.5. **a** Staining for alkaline phosphatase activity (ALPL, red) in preosteoblasts and osteoblasts for wild type (WT) and mutant (*Hhip*^*−/−*^) coronal sutures. f frontal bone; p parietal bone, sm suture mesenchyme. **b** Immunohistochemistry for RUNX2 (green). Sections are the same as shown in **a**. **c** Immunohistochemistry for SP7 (green). **d** Proliferation detected by EdU incorporation (green). Sections are counterstained for ALPL activity (red) and DAPI (blue). **e** Quantification of EdU incorporation in osteogenic fronts (OF) and suture mesenchyme (SM). WT and *Hhip*^*−/−*^ are indicated in black and red, respectively. *n* = 3 WT and three *Hhip*^*−/−*^. **f** Quantification of cell numbers per section in OFs and SM. *n* = 3 WT and three *Hhip*^*−/−*^. **g** TUNEL assay for apoptotic cells. Inset, example of an apoptotic cell in non-sutural tissue on the same slide. White dashed outlines, frontal and parietal bones. Data in **e** and **f** are presented as dot plots showing mean ± standard deviation. Results presented in **a**, **b**, **c**, **d**, and **g** are representative of *n* = 3 WT and three *Hhip*^*−/−*^ independent samples. Sections are in the transverse plane. Scale bar, 50 μm. Source data are provided as a Source Data file.

We reasoned that *Hhip* expression in the SM is necessary for normal coronal suture development and loss of its function may lead to suture dysgenesis. Thus, we examined the coronal suture in *Hhip*^*−/−*^ mice and found a previously unreported coronal suture defect. At E16.5, staining for ALPL activity showed that the overlap of frontal and parietal bones seen in the WT was reduced or absent in mutant sutures so that the OFs are more closely apposed (Fig. 5a). In the WT suture, RUNX2, a marker of osteoprogenitors and more differentiated osteoblasts, was expressed in the SM, OFs, and more differentiated osteoblasts (Fig. 5b) while SP7, a marker of committed preosteoblasts, was expressed in the OFs and more differentiated osteoblasts (Fig. 5c). RUNX2 and SP7 expression in mutant sutures localized to the same sutural regions as in WT, despite the altered morphology of the bones (Fig. 5b, c). No difference in proliferation of frontal or parietal OFs or SM was seen between WT and mutant sutures (Fig. 5d, e). Total cell numbers in the frontal and parietal OFs were similar between WT and mutant sutures, but there was a trend toward lower cell numbers in mutant SM (Fig. 5f). Mutant OFs appeared broader than the more tapered WT OFs (Fig. 5a–c). To quantify this broadening, the area of OF ALPL activity within 50 μm of the edge of frontal and parietal bones was measured. Mutant frontal OFs showed no difference compared to WT (1193 ± 184 μm^2^, *n* = 3, compared to 1282 ± 216 μm^2^, *n* = 3, *p* = 0.62) but mutant parietal OFs had a significantly greater area compared to WT (1207 ± 45 μm^2^, *n* = 3, compared to 723 ± 8 μm^2^, *n* = 3, *p* = 0.000054). Assessment of apoptosis by the TUNEL assay found no apoptotic cells in WT or mutant sutures (Fig. 5g).

**Fig. 6:**
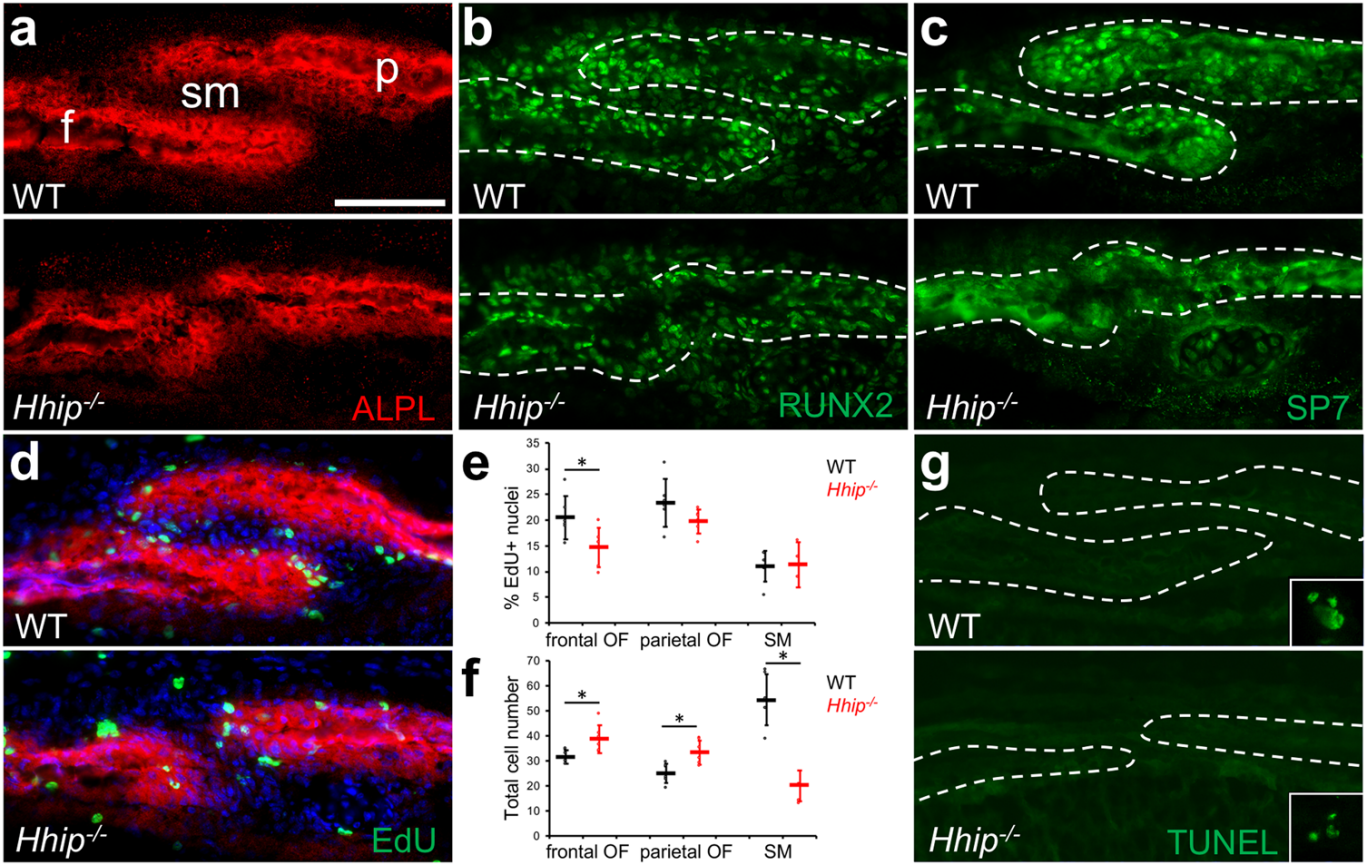
Coronal suture dysgenesis in *Hhip*^*−/−*^ mice at E18.5. **a** Staining for alkaline phosphatase activity (ALPL, red) in preosteoblasts and osteoblasts for WT and *Hhip*^*−/−*^ coronal sutures. f, frontal bone; p, parietal bone; sm, suture mesenchyme. **b** Immunohistochemistry for RUNX2 (green). Sections are the same as shown in **a**. **c** Immunohistochemistry for SP7 (green). **d** Proliferation detected by EdU incorporation (green). Sections are counterstained for ALPL activity (red) and DAPI (blue). **e** Quantification of EdU incorporation in osteogenic fronts (OF) and suture mesenchyme (SM). WT and *Hhip*^*−/−*^ are indicated in black and red, respectively. Asterisk indicates a significant difference using the two-tailed Student’s *t*-test (*P* = 0.030). *n* = 6 WT and six *Hhip*^*−/−*^. **f** Quantification of cell numbers per section in OFs and SM. Asterisk indicates a significant difference using the two-tailed Student’s *t*-test (*P* = 0.018, 0.008, and 0.00004, respectively). *n* = 6 WT and six *Hhip*^*−/−*^. **g** TUNEL assay for apoptotic cells. Inset, example of an apoptotic cell in non-sutural tissue on the same slide. White dashed outlines, frontal and parietal bones. Data in **e** and **f** are presented as dot plots showing mean ± standard deviation. Results presented in **a**, **b**, **c**, and **g** are representative of *n* = 3 WT and three *Hhip*^*−/−*^ independent samples. Sections are in the transverse plane. Scale bar, 100 μm. Source data are provided as a Source Data file.

At E18.5 the abnormal *Hhip*^*−/−*^ phenotype was more severe. Coronal sutures still had little or no overlap of frontal and parietal bones along the length of the suture, and in some regions ALPL expression showed that the OFs were so closely apposed that there was little or no intervening SM (Fig. 6a). RUNX2 expression in mutant sutures localized to the same sutural regions as in WT (Fig. 6b). Where the mutant phenotype was more pronounced, SP7 expression was lower and less distinct between the OFs and presumptive SM than in WT sutures (Fig. 6c). A trend toward decreased preosteoblast proliferation in mutant OFs compared to WT was seen but only reached significance in the frontal OF (Fig. 6d, e). Total cell numbers in the mutant frontal and parietal OFs were significantly increased compared to WT, while there was a significant decrease in cell numbers in mutant SM (Fig. 6f). The increase in mutant OF cell numbers was associated with an apparent broadening of mutant OFs compared to WT. To quantify this broadening, the area of OF ALPL activity within 50 μm of the edge of frontal and parietal bones was measured. Mutant frontal OFs showed a trend of greater area compared to WT (2053 ± 411 μm^2^, *n* = 6, compared to 1562 ± 381 μm^2^, *n* = 6, *p* = 0.06), and mutant parietal OFs had a significantly greater area compared to WT (1773 ± 281 μm^2^, *n* = 6, compared to 1231 ± 355 μm^2^, *n* = 6, *p* = 0.02). Assessment of apoptosis by the TUNEL assay found no apoptotic cells in WT or mutant sutures (Fig. 6g). At P0, a narrow region of cells expressing ALPL activity between frontal and parietal bones could be found in the most severely affected regions of the coronal suture (Supplementary Fig. 7). However, neonatal lethality of *Hhip*^*−/−*^ mice due to lung hypoplasia precluded observation of later phenotypes^14^. *Hhip* clearly was required for normal coronal suture development.

**Fig. 7:**
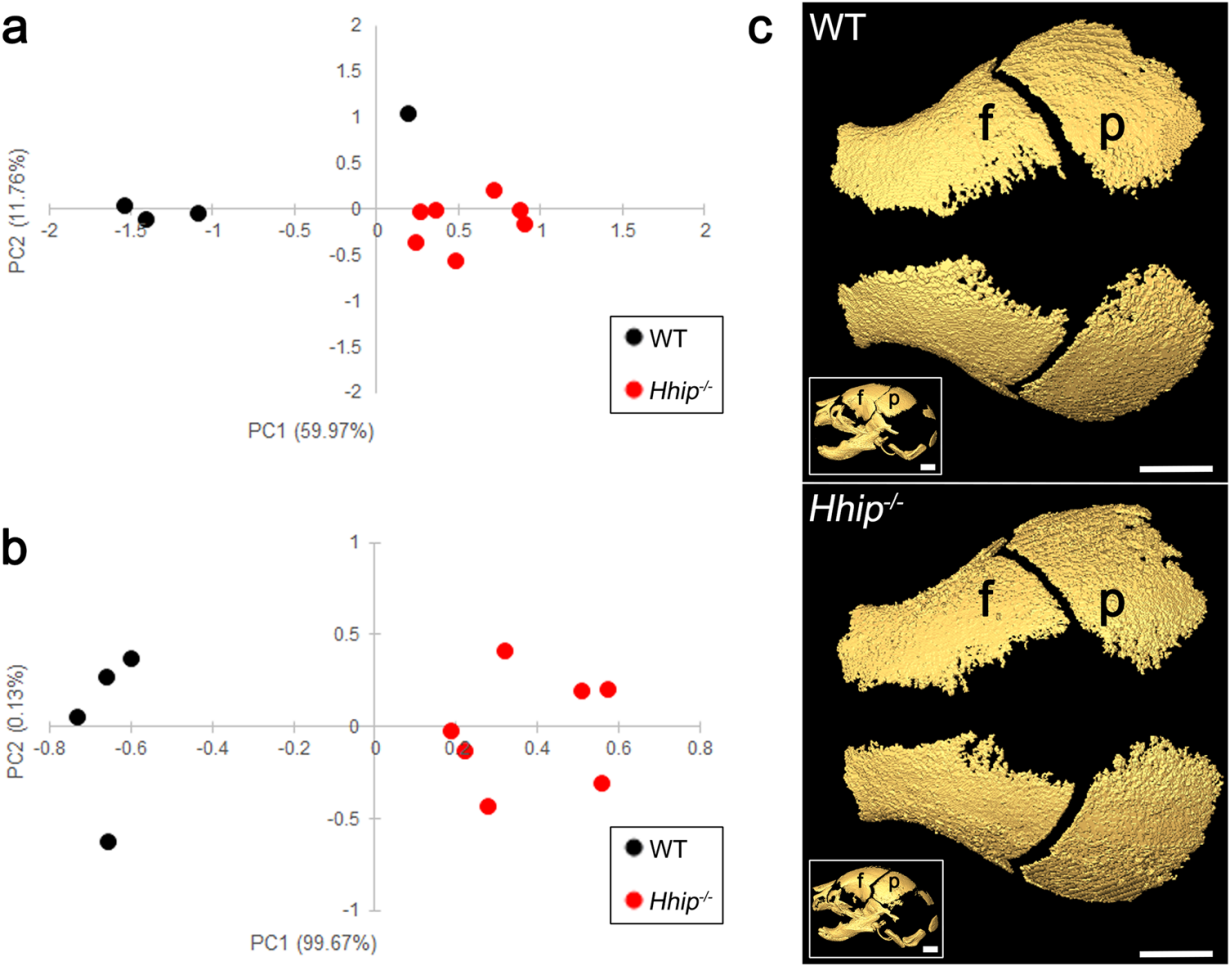
Results of principal components analyses (PCA) of data from microcomputed tomography (microCT) imaging of *Hhip*^*−/−*^ heads at E18.5. Scatter plots of results of PC1 and PC2 scores based on PCA analysis of unique linear distances among 3D landmarks for **a** form and **b** shape (adjusting for the effects of allometry) of WT (*n* = 4) and *Hhip*^*−/−*^ (*n* = 7) skulls. **c** 3D reconstruction of microCT images showing dorsal views of the frontal (f) and parietal (p) bones of WT and *Hhip*^*−/−*^ calvaria. Insets show lateral views of the whole skull. Images are representative *n* = 4 WT and seven *Hhip*^*−/−*^ skulls. Scale bars, 1 mm.

The morphology of WT and *Hhip*^*−/−*^ calvaria was compared at E18.5 by microcomputed tomography (microCT) using the three-dimensional (3D) coordinates of the locations of 39 anatomical landmarks (Supplementary Table 2). Principal component analysis showed that WT and mutant calvaria segregated by both form (size and shape, Fig. 7a) and shape (normalized for size, Fig. 7b) according to genotype. The mineralization fronts of frontal and parietal bones within all WT and mutant coronal sutures were separated and not fused (Fig. 7c).

### Population mapping of *Hhip*^*−/−*^ coronal sutures identifies a positional change in the *Mmp13*-expressing osteoblast population

**Fig. 8:**
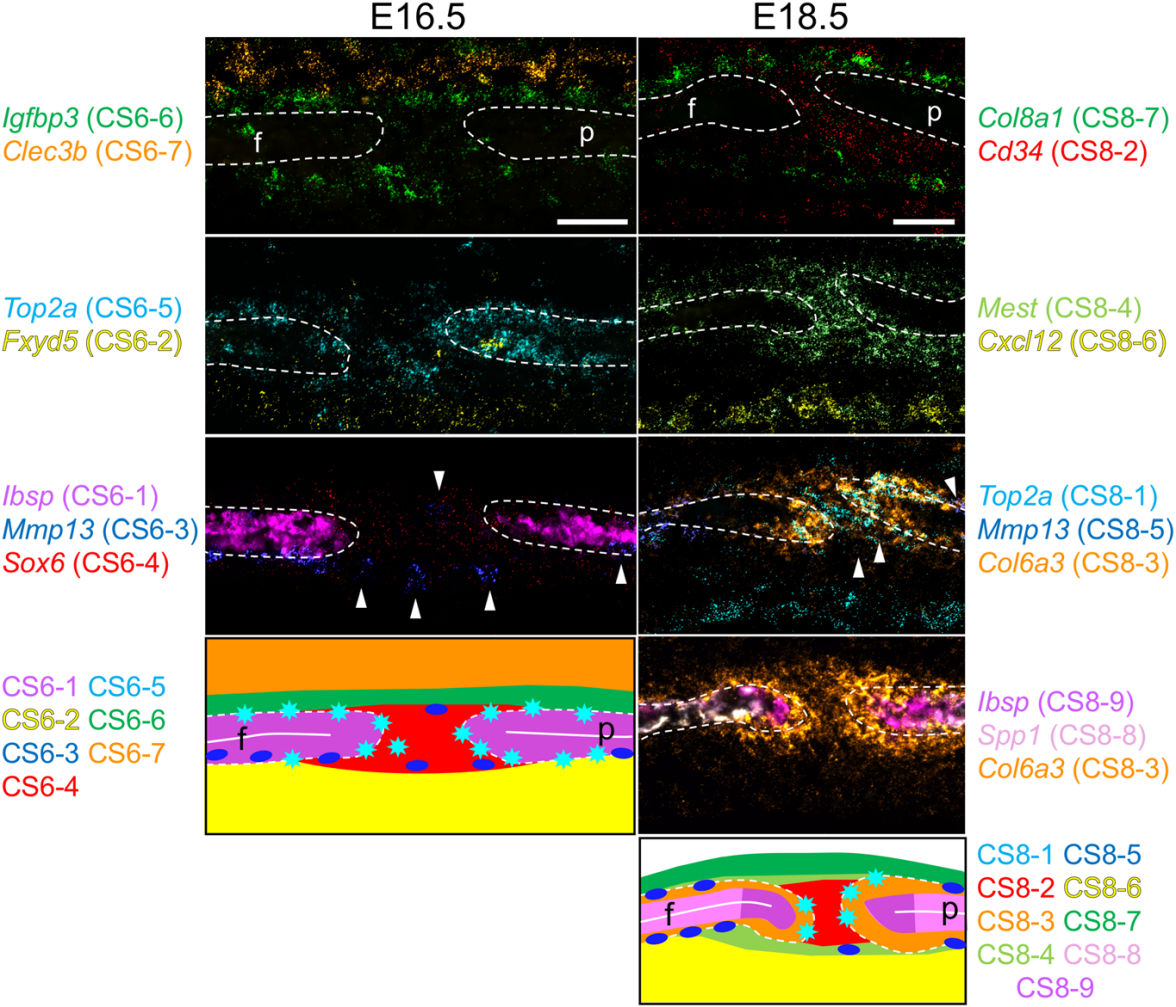
Localization of *Hhip*^*−/−*^ coronal suture populations by smFISH. Each pseudo-colored panel shows an individual section hybridized with probes for the indicated genes at each age. Expression of the E18.5 CS8-2 marker, *Cd34*, can be compared to WT in Supplementary Fig. 3a. Schematics summarize the spatial distribution of populations, color-coded as in smFISH. White arrowheads indicate novel *Mmp13* expression. Dashed outlines indicate frontal (f) and parietal (p) bones; white horizontal lines indicate osteoid. Sections are in the transverse plane. smFISH was performed on three independent samples with similar results. Scale bars, 50 μm.

To assess cell population changes in *Hhip*^*−/−*^ coronal sutures we mapped the expression of the WT population markers at E16.5 and E18.5 (Fig. 8 and Supplementary Figs. 8 and 9). As *Hhip* is not expressed in *Hhip*^*−/−*^ mice we used expression of *Cd34* as a marker of CS8-2. At both ages most population markers showed similar relative expression patterns compared to WT (Figs. 1c and 2c). *Cd34* expression remained present between frontal and parietal OFs at E18.5 but its restricted domain clearly reflected the loss of bone overlap in the mutant coronal suture (Fig. 8 and Supplementary Figs. 3a and 9). The most notable difference was in the position of the *Mmp13*-expressing population. At both ages *Mmp13*-expressing cells were found closer to the *Hhip*^*−/−*^ parietal OF and present in the SM in contrast to the WT (Fig. 8 and Supplementary Figs. 8 and 9).

### Hedgehog signaling is altered in *Hhip*^*−/−*^ coronal sutures

**Fig. 9:**
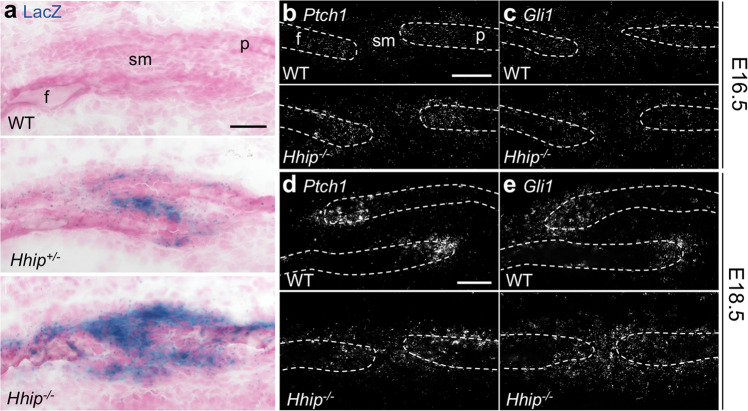
Altered HH signaling in the *Hhip*^*−/−*^ coronal suture. **a** LacZ staining (blue) in WT, *Hhip*^*+/−*^, and *Hhip*^*−/−*^ coronal sutures at E18.5, counterstained with nuclear fast red. **b**–**e** smFISH for **b**, **d** *Ptch1*, and **c**, **e** *Gli1* in WT and *Hhip*^*−/−*^ coronal sutures at **b**, **c** E16.5 and **d**, **e** E18.5. Dashed outlines indicate frontal (f) and parietal (p) bones; sm, suture mesenchyme. Sections are in the transverse plane. Results presented in **a** are representative of *n* = 2 WT, two *Hhip*^*+/−*^, and two *Hhip*^*−/−*^ independent samples. Results presented in **b**–**e** are representative of *n* = 3 WT and three *Hhip*^*−/−*^ independent samples. Scale bars, 50 μm.

We next investigated changes in the expression of downstream HH pathway components. The *Hhip* mutant locus contains a LacZ reporter, present in one copy in *Hhip*^*+/−*^ mice and two copies in *Hhip*^*−/−*^ mice^14^. Expression of this reporter at E18.5 was increased disproportionately in homozygous compared to heterozygous mutant coronal sutures and was more widely expressed, indicating that HH signaling at the *Hhip* locus was increased in *Hhip*^*−/−*^ coronal sutures (Fig. 9a). We next assessed the expression of *Ptch1* and *Gli1*. In WT sutures at E16.5, *Ptch1* expression was highest in OFs but extended into adjacent mesenchyme and SM at lower levels (Fig. 9b). *Gli1* expression overlapped *Ptch1* but extended further into the adjacent mesenchyme and throughout the SM (Fig. 9c). In WT sutures at E18.5, *Ptch1* expression was highly enriched in the OFs and absent from SM (Fig. 9d). *Gli1* expression was enriched in the OFs and extended a short distance into the surrounding mesenchyme (Fig. 9e) but was absent in the SM where *Hhip* expression was highest (Fig. 2c). In mutant sutures at E16.5, *Ptch1* and *Gli1* expression were similar to WT (Fig. 9b, c). In mutant sutures at E18.5, expression levels of *Ptch1* and *Gli1* were not notably altered (Fig. 9d, e), but their expression now extended throughout the SM between the closely apposed OFs.

In addition to the HH pathway, coronal suture formation is regulated by many other signaling pathways. The FGF pathway is the pathway most frequently affected by mutations in syndromic craniosynostosis involving the coronal suture. TGF pathway mutations also result in coronal suture fusion^7,8^. IHH signaling may also upregulate BMP signaling^31^. To determine whether these pathways are affected in the *Hhip*^*−/−*^ coronal suture we performed immunohistochemistry for phosphoproteins indicative of pathway activation. No difference was seen between the level or relative location of phospho-ERK1/2 and phospho-p38 (FGF signaling), phospho-SMAD2 (TGF signaling), or phospho-SMAD1/5/9 (BMP signaling) between WT and *Hhip*^*−/−*^ coronal sutures at E18.5 (Supplementary Fig. 10).

## Discussion

Using single-cell with bulk RNA-seq analysis we have better defined the distinctive composition of the coronal suture at the transcriptional and cell population levels. By scRNA-seq we identified five and eight major cell populations with mesenchymal or osteoblast identities present at E16.5 and E18.5, respectively; these do not include hypodermis or dura mater populations. There were five populations common to both ages: *Hhip*-expressing SM (CS6-4 and CS8-2), ectocranial SM (CS6-6 and CS8-7), proliferating preosteoblasts at both frontal and parietal OFs (CS6-5 and CS8-1), and differentiated *Ibsp*- and *Mmp13*-expressing osteoblasts (CS6-1 and CS8-9; CS6-3 and CS8-5, respectively). The differences between the two ages were the addition at E18.5 of one mesenchyme population and two osteoblast populations. The mesenchyme between the overlapping frontal and parietal bones (CS8-2) was distinguished from the mesenchyme extending beyond this overlap, adjacent to osteogenic fronts (CS8-4). Within osteoblast populations, a distinct periosteal population (CS8-3) and a more differentiated *Spp1*-expressing population were present (CS8-8).

Populations found in the frontal and parietal bones potentially may differ in their gene signatures because of the division of the coronal suture between the frontal bone derived from neural crest and the parietal bone and SM derived from mesoderm. By comparing the spatial locations of populations with respect to the known boundary of neural crest and mesoderm, we found that the *Hhip*-expressing SM populations (CS6-4, CS8-2, and CS8-4) and ectocranial mesenchyme (CS6-6 and CS8-7) are located in tissue of the mesoderm lineage. The possibility of lineage-specific expression differences also is suggested by previous studies showing that neural crest has increased osteogenic capacity compared to mesoderm. Postnatal frontal bone has an enhanced osteogenic capacity compared to the parietal^32,33^, and embryonic and postnatal frontal bone has increased expression of pro-osteogenic FGF ligands and receptors compared to the parietal^34^. Our analysis identified such a difference in populations between frontal and parietal bones. This was the presence of an *Mmp13*-expressing osteoblast population principally on the endocranial surface of the frontal bone (CS6-3 and CS8-5), which was not present on the parietal bone near the suture. This could represent a lineage-specific difference. However, this may be a difference in bone formation processes between frontal and parietal bones in the suture region because *Mmp13*-expressing osteoblasts, known to be involved in bone remodeling^35,36^, were present in more mature regions of both bones away from the suture. Expression of *Mmp13* also has been reported in the human embryonic calvaria in more mature endocranial and trabecular bone^37^. Indeed, we did not identify lineage-specific distinctions for other populations common to neural crest- and mesoderm-derived tissue. We found only one proliferating osteoblast population that localizes to both OFs at each age (CS6-5 and CS8-1). Similarly, the *Ibsp*-, *Spp1*-, and *Postn*/*Col6a3*-expressing osteoblast populations (CS6-1, CS8-9, CS8-8, and CS8-3) are present in frontal and parietal bones.

Many genes have been identified that cause craniosynostosis when mutated in humans. In the coronal suture populations that we identified, expression of such genes was enriched in the ectocranial mesenchyme population at both ages (CS6-6 and CS8-7) and the periosteal (CS8-3) and *Ibsp*-expressing (CS8-9) osteoblast populations at E18.5, suggesting that genetic dysregulation within various cell types can lead to suture fusion. Significant enrichment of craniosynostosis gene expression also was found in the *Mmp13*-expressing osteoblast (CS6-3 and CS8-5) population. As this population was located in more mature bone away from the SM and OFs, some caution must be used in interpreting this particular finding. At one or both ages, this population expresses the craniosynostosis-related genes *Alpl*, *Fgfr2*, and *Fgfr3* as marker genes. While these genes may be more highly expressed in the *Mmp13*-expressing osteoblast population in comparison to others, these genes also are expressed in the osteoblast populations closer to and within the OFs. Mutations in these genes therefore may result in craniosynostosis through their action in more than one or other cell populations. In addition, we have assessed the coronal suture populations at E16.5 and E18.5, but mutated genes may act on other cell populations to perturb suture development if expressed at earlier stages.

We noted differences between the coronal and other calvarial sutures. Coronal suture cell populations differed from the frontal suture, which we previously characterized by single-cell and bulk RNA-seq^13^. The major differences were between the SM populations of each suture. *Hhip* expression was a feature of coronal SM at E16.5 and E18.5, while its expression was enriched in OFs in the frontal suture at these ages. The coronal ectocranial mesenchyme expressed genes such as *Col8a1* and *Igfbp3*. The frontal suture contained a population with a similar gene expression profile that also extended ectocranially over the frontal bones, but in contrast to the coronal suture it comprised the majority of the frontal SM. A mesenchymal population in the frontal suture, enriched for expression of *Acta2* and other genes involved in cell contractility or tendon and ligament development, was not identified in the coronal SM. This suggests differences in the mechanical environment of the frontal suture, in which frontal bones are apposed end-to-end, and the coronal suture, in which frontal and parietal bones overlap. This differing morphology may be influenced by or shape responses to potential differences in tension forces at each suture during expansion of the skull and brain. Finally, the *Mmp13*-expressing osteoblast population was not identified in the frontal suture, but this may represent a difference in the extent of differentiation processes at each suture.

Our neonatal tracing studies of the *Hhip-*CreERT2-expressing SM revealed interesting features of the relationship between CS8-2 and other coronal suture populations. Previous *ex utero* labeling studies of the embryonic frontal and parietal bone primordia suggested that bone growth towards the skull apex is sustained by the proliferation of osteoprogenitors within the OFs with minor recruitment of adjacent midline mesenchymal cells, but these studies did not directly target coronal SM cells^10,38^, whose fate was otherwise undetermined. Trajectory analysis of our single-cell populations did not place CS8-2 within a direct line of differentiation through proliferating osteoprogenitors, periosteal cells, and osteoblasts. Indeed, *Hhip-*CreERT2-labeling showed that descendant cells do not appear to contribute directly to frontal and parietal bone growth during approximately the first month of postnatal development, during which time they may function to maintain separation of the frontal and parietal bones, and only become incorporated appreciably into bone after this period. Various recent studies have identified adult suture stem cells (SSCs) that develop postnatally within the SM of all calvarial sutures and are necessary to maintain and repair calvarial bone^39–47^. The earliest age at which these stem cells have been suggested to be present is P6 in the sagittal suture, when slow-cycling cells, indicative of potential stem cells, have been identified^40^. Expression of *Axin2* and *Gli1*, which mark SSC populations, is enriched centrally in SM by P9 and P14, respectively^39,40,42^. It is therefore likely that descendants of neonatal, *Hhip*-expressing cells contribute to the SSC population that develops in the postnatal coronal SM, and thereafter are able to contribute directly to calvarial bone growth.

HH signaling plays an important role in embryonic suturogenesis, where it is believed to have pro-osteogenic functions in the OFs and SM^48^. *Ihh* is expressed in OFs^49,50^ and appears to be the only functional HH ligand during calvarial development^39,51^. In *Ihh*^*−*/*−*^ calvaria, intramembranous ossification is decreased resulting in wider sutures^51–54^. In humans and mice, duplication of regulatory elements driving *Ihh* expression results in fusion of various sutures including the coronal^55–57^. Mutations in other HH pathway members such as *Ptch1*, *Smo*, *Gli3*, and *Rab23* also cause craniosynostosis in humans and/or mice^58–61^. HH signaling also is important for the function of the SSC population in the maintenance and repair of calvarial bone^39,42^.

*Hhip* expression in SM populations (CS6-4 and CS8-2) suggests that inhibition of functional HH signaling is required in SM at least in later embryonic stages for normal suturogenesis. Indeed, we found that homozygous loss of *Hhip* results in altered coronal suture morphology by E16.5 and depletion of SM at E18.5 and P0, so that OFs were closely apposed or even bridged by osteogenic cells expressing ALPL. We did not find differences in apoptosis between WT and mutant sutures to explain this defect. Decreased proliferation was seen only in the frontal OF at E18.5, suggesting that it was not a direct effect of altered HH signaling but is consistent with a loss of osteoprogenitors in favor of osteogenic differentiation, as is the broadening of osteogenic fronts between E16.5 and E18.5. HH signaling as measured by expression of the LacZ reporter at the *Hhip* locus was increased in *Hhip*^*−/−*^ SM, consistent with loss of inhibition by HHIP. Importantly, *Gli1* expression, which may represent the extent of osteogenic HH signaling, now extended throughout the remaining mutant SM. In addition, no change in the activity of FGF, TGF, or BMP pathways was noted in *Hhip*^*−/−*^ coronal sutures. Taken together, this suggests that the loss of spatial restriction of HH signaling in *Hhip*^*−/−*^ sutures deregulates osteoprogenitor recruitment from the SM leading to loss of SM and closer approximation of the OFs.

During embryonic suture development there is essentially no mixing between neural crest and mesoderm lineages within the coronal suture^9–11^. Osteoprogenitors forming the neural crest-derived frontal bone must be restricted to the region of neural crest-derived mesenchyme at the OF. In contrast, the mesoderm-derived parietal bone and SM are contiguous, and so the SM, which expresses the master osteogenic transcription factor RUNX2, potentially provides a large pool of osteoprogenitors along the parietal bone surface. However, the presence of similar proliferating osteoprogenitor populations at the frontal and parietal OFs suggests a common mechanism for spatially limiting recruitment of osteoprogenitors to the OFs that is independent of lineage. This mechanism could in part consist of IHH expressed by osteoblasts at the OFs (CS6-1 and CS8-9) acting on an adjacent pool of osteoprogenitors (CS6-5 and CS8-1). Osteoprogenitor recruitment from the SM between the frontal and parietal bones would be inhibited by the presence of HHIP to maintain a non-ossifying SM.

The source of osteoprogenitors during embryonic and early postnatal growth may lie within the *Gli1*-expressing cells adjacent to the OFs. The descendants of *Gli1*-Cre-expressing cells labeled and traced from E13.5 occupied both the SM and calvarial bones at P60^62^. However, *Gli1* expression potentially spans osteoblasts, OFs, and adjacent SM, and the extent of *Gli1* expression at E13.5 or the region of origin of labeled cells was not determined in that study. As postnatal growth continues, *Gli1*-expressing cells arise in the central mesenchyme as part of the SSC population^39,42^.

Another study analyzed the WT, murine coronal suture by scRNA-seq at earlier stages of E15.5 and E17.5, and the dissections included the hypodermis and meninges^20^, which were largely excluded from our study. Within the isolated suture tissue common to both studies, similar proliferating, mesenchymal, and osteoblast populations were identified. Both studies compared the transcriptome of the coronal suture with our previously published transcriptome data of the frontal suture^13^ and identified distinctions between the two sutures. Importantly, our study differed in the identification of enriched *Hhip* expression in a specific mesenchyme population, CS8-2. In their study^20^, *Hhip* was a marker of a proliferative population (PO1), rather than the progenitor population (OG1) that corresponds to our CS8-2 population. Various factors may contribute to differences of identified marker genes within populations between the two studies. These include differences in the extent of inclusion of bone adjacent to coronal suture and presence or absence of hypodermal and meningeal tissues, which may alter the ranking of marker genes in comparing smaller or larger numbers of individual populations. Also, differences in the number of cells analyzed and in the parameters used for bioinformatic analysis may alter relative gene expression among populations.

In conclusion, we define transcriptionally cell populations of the murine coronal suture and their constituent marker genes during embryonic development using scRNA-seq. We found that enriched *Hhip* expression was a feature of a specific SM population distinguishing the coronal suture from other calvarial sutures. This discovery led us to identify a coronal suture phenotype in *Hhip*^*−/−*^ mice, in which potential osteoprogenitors are depleted from the SM. Descendants of the neonatal *Hhip*-expressing population do not appear to contribute directly to bone growth during the first month of postnatal development, but after this period can be incorporated into the frontal and parietal bones. We propose a revised view of the role of HH signaling during coronal suturogenesis in which functional HH signaling is excluded from the majority of the SM by HHIP to spatially restrict osteoprogenitor induction and recruitment during embryonic and early postnatal development, preserving the SM as a barrier between the frontal and parietal bones. Our transcriptomic approach greatly expands opportunities for hypothesis-driven research in coronal and other suture development.

## Methods

### Mice

Mouse procedures were in compliance with animal welfare guidelines mandated by the Institutional Animal Care and Use Committee (IACUC) of the Icahn School of Medicine at Mount Sinai and the Pennsylvania State University. Timed matings of C57BL/6J mice (The Jackson Laboratory, 000664), heterozygous *Hhip*^*tm1Amc*^/J mice (The Jackson Laboratory, 006241; on a mixed C57BL/6, Swiss-Webster, 129 background; homozygotes referred to as *Hhip*^*−/−*^), or heterozygous *Hhip*-EGFP_T2A_CreERT2 mice (referred to as *Hhip*-CreERT2; European Mouse Mutant Archive, 12335; on a mixed C57BL/6NTac and C57BL/6J background) and homozygous Ai14 mice (The Jackson Laboratory, 007914; on a C57BL/6J background) were performed to obtain embryos or postnatal mice at the required ages. Water and food were available ad libitum, and mice were maintained on a 12:12 h light:dark cycle at a temperature of 20–22° Celsius and 30–70% humidity. Genotyping was performed by polymerase chain reaction of tail DNA. Sex genotypes were identified as described previously^63,64^. *Hhip* genotypes were identified as described by The Jackson Laboratory. *Cre* genotypes were identified using previously published primers MG-cre800 and MG-cre1200^65^.

### Tamoxifen induction and fate mapping

Tamoxifen (Sigma-Aldrich, T5648) was dissolved in corn oil (Sigma-Aldrich, C8267) at a concentration of 20 mg/ml. *Hhip*-CreERT2 activity was induced in pups by intraperitoneal injection of lactating dams with 2 mg (100 μl) of tamoxifen solution on the days specified and chased for the length of time indicated. Calvaria were dissected, fixed in 4% paraformaldehyde (PFA) overnight, and demineralized in 10% EDTA for up to 3 weeks depending on age, equilibrated in phosphate-buffered saline (PBS)/30% sucrose, and embedded in Tissue-Tek Optimal Cutting Temperature (OCT) compound (Sakura, 4583). Calvaria were sectioned at 10 μm, stained for alkaline phosphatase (ALPL) activity with p-nitroblue tetrazolium chloride/5-bromo-4-chloro-3-indolyl phosphate (NBT/BCIP) (Sigma-Aldrich, 11681451001) by standard methods and counterstained with DAPI. ALPL staining was imaged by brightfield microscopy and tdTomato-expressing cells and DAPI-stained nuclei were imaged by fluorescent microscopy. Compound images were created in Adobe Photoshop by inverting the ALPL brightfield image and converting it to a green channel in combination with tdTomato (red channel) and DAPI (blue channel) images.

### scRNA-seq library preparation

Coronal sutures, encompassing the SM, OFs, and frontal and parietal bones in the region of overlap, were dissected from C57BL/6J mice (Supplementary Fig. 1a). Ectocranial and endocranial membranes were removed to the extent possible, and frontal and parietal bones were separated with forceps to expose the SM. Sutures of both sexes were combined for processing. Two libraries each at E16.5 and E18.5 were created. The E16.5 libraries each consisted of pooled sutures from 13 embryos obtained on the same day; the E18.5 libraries each consisted of pooled sutures from eight and 13 embryos obtained on separate days. Pooled E16.5 sutures were digested at 37 °C in αMEM (Gibco, 32571-036) with 0.2% collagenase type II (Worthington, LS004176), 0.2% dispase II (Sigma-Aldrich, 4942078001), and 1 U/μl DNase (Qiagen, 79254). Sutures were serially digested with agitation five times for 10 min each in a shaking incubator. Successive fractions were pooled on ice with the addition of FBS to 2%. Cell suspensions were strained through a 40 μm filter (Falcon, 352340), pelleted at 400 g for 7 min, washed in PBS/1% BSA, and resuspended in PBS/1% BSA. Pooled E18.5 sutures were similarly processed and red blood cell lysis (Miltenyi Biotec, 130-094-183) was performed after pelleting filtered cells. scRNA-seq 3' expression libraries were prepared on a Chromium instrument (10X Genomics, model GCG-SR-1) using the Chromium Single Cell Gene Expression kit (version 3 for E16.5 libraries; version 2 for E18.5 libraries) by the Technology Development Facility at the Icahn School of Medicine at Mount Sinai. Sequencing was conducted by the Genetic Resources Core Facility, Johns Hopkins Institute of Genetic Medicine. Libraries were first run on an Illumina MiSeq at 26x8x98 and analyzed to confirm the number of captured cells and assess capture efficiency prior to sequencing. E16.5 libraries were sequenced in a single pool on an Illumina NovaSeq S1. E18.5 libraries were sequenced separately on an Illumina HiSeq 2500. Sequencing used standard Illumina primers, where read 1 was the UMI, read 2 was the library index, and read 3 was the transcript. E16.5 libraries consisted of a final number of 941 and 1185 cells. E18.5 libraries consisted of a final number of 2625 and 1624 cells.

### scRNA-seq analysis

Preprocessing of 10X Genomics scRNA-seq data, clustering, and cell type identification was performed using 10X Genomics Cellranger v3 software as previously described with a few modifications^13^. Cells with >800 genes detected, and genes detected in >5 cells were included. Cells with outlier numbers of detected genes and/or high expression of mitochondrial genes were detected using mvoutlier R package v2.0.8^66^ and removed. The cells from both replicates and both embryonic days were normalized using regularized binomial negative regression^67^ and integrated based on common transcripts or ‘anchors’^68^. The cleaned and integrated data contained 19,903 genes and 5142 cells across all samples, and was then used for initial clustering. Thirteen principal components (PCs) were used to perform unsupervised shared nearest neighbor graph-based clustering (*k* = 100) as implemented in the package Seurat v3.1.1^68,69^. Differential gene expression analysis among cell clusters and stages was tested by logistic regression using replicate as a latent variant, an FDR ≤ 0.05 and lnFC expression threshold ≤0.25. Mitochondrial and erythroid-specific gene expression were treated as covariates as previously described^13^.

### Identification of populations within the coronal suture

To analyze the relationship between the SM, osteoblasts, and adjacent populations in the initial UMAP plot (Supplementary Fig. 1b), we performed a second unsupervised clustering (*k* parameter = 50, resolution = 0.8) and differential gene expression analysis for these cell types separately for each embryonic day, using the 13 and 15 top PCs for E16.5 and E18.5 respectively. In addition, we used a combined Fisher *p*-value of 0.01 and a minimum lnFC threshold of 0.1 to determine significant markers^70^.

### Gene ontology enrichment analyses

Gene ontology (GO) biological process (BP), molecular function (MF), and/or cellular component (CC) enrichment analyses for each single-cell cluster were performed using the gProfileR v0.6.4 package^71^ as previously described^13^.

### Gene enrichment analysis

Lists of 96 human and 31 mouse genes associated with craniosynostosis and subsets of genes associated with coronal synostosis^5,7,25,26^ (Supplementary Dataset 2) were tested for significant (*p* ≤ 0.05) gene set enrichment against the single-cell CS populations, determined by scRNA-seq analysis using Fisher’s exact test and using Bonferroni correction for multiple comparisons. This method also was used to match the single-cell CS populations identified at E16.5 with those identified at E18.5.

### Comparative analysis of calvarial suture RNA-seq data

To compare gene expression profiles of calvarial sutures we leveraged RNA-seq data that we generated for SM and OF regions of 11 craniofacial sutures as part of the Transcriptome Atlases of the Craniofacial Sutures FaceBase2 project^27,28^. Briefly, count-per-million (CPM) values for 629 SM and OF RNA-seq libraries for coronal, frontal, sagittal, lambdoid, intermaxillary, internasal, interpremaxillary, interpalatine, maxillary-palatine, premaxillary-maxillary, and squamoparietal sutures at E16.5 and E18.5 (up to five replicates each) were filtered to retain genes with ≥1 CPM in more than 10% of samples, and then normalized using the weighted trimmed mean of M-values (TMM) method^72^. Finally, we applied the Voom transformation^72^ to the data, which included transforming the matrix to log2 CPM data, estimating the mean-variance relationship, and computing appropriate observation-level weights using this relationship. Differential gene expression analysis was then performed between the OF and SM regions of the calvarial sutures as described previously^13^.

### Cell communication analysis

Cell communication analysis was performed with CellPhoneDB v2.0 on the scRNA-seq suture-specific populations at the E18.5 stage^29^. Physical interactions with IHH were downloaded from String, manually curated, and added to the CellPhoneDB default database.

### Monocle3 analysis

Trajectory analysis with UMAP and approximate graph abstraction with Monocle3 was performed for the CS populations of the E18.5 data^73^. For this analysis, cell cycle related genes (GO:0007049) were removed. The trajectory and pseudotime estimates were inferred, using ten dimensions and setting the CS8-2 population as the root state.

### Histochemical staining

Alkaline phosphatase (ALPL) staining with fast red TR (Sigma-Aldrich, F8764) was performed as described previously^13^. Nuclei were counterstained with DAPI. LacZ staining was performed by standard methods and sections counterstained with nuclear fast red (Vector Laboratories, H-3403).

### Immunohistochemistry and cytochemistry

Immunohistochemistry and EdU staining were performed on 10 μm sections from either fresh frozen or 4% PFA-fixed cryoembedded heads prepared as previously described^74^. Antibody staining for RUNX2 (1:200; rabbit anti-RUNX2, Sigma-Aldrich, HPA022040) and SP7 (1:500; rabbit anti-SP7/Osterix, Abcam, ab22552) was performed after ALPL staining using standard procedures, and primary antibodies were detected with donkey anti-rabbit IgG Alexa Fluor 488 (1:400; Thermo Fisher Scientific, A-21206). Antibody staining for phospho-P42/44 MAPK (ERK1/2) (1/100; Cell Signaling Technology, 4376), phospho-p38 MAPK (1/200; Cell Signaling Technology, 4631), phospho-SMAD2 (1/100; Cell Signaling Technology, 3108), and phospho-SMAD1/5/9 (1/100; Cell Signaling Technology, 9511) was performed on fresh frozen sections without antigen retrieval, and primary antibodies were detected with goat anti-rabbit IgG Alexa Fluor Plus 555 (1:400; Thermo Fisher Scientific, A-32732). For EdU quantification, pregnant mice were injected with EdU (250 μg/10 kg body weight) 2 h before sacrifice. EdU staining was performed with the Click-iT Plus EdU Alexa Fluor 488 Imaging Kit (Thermo Fisher Scientific, C10637) as described by the manufacturer. Sections were counterstained with DAPI. EdU-positive nuclei and total nuclei were counted within the OFs and SM of five non-consecutive sections per coronal suture and averaged. In OFs nuclei were counted within the ALPL-positive domain between the SM and the start of the osteoid, or within a 50 μm distance if the osteoid was not apparent. SM was defined as ALPL-negative cells between the ends of the ALPL-positive OFs. OF thickness was compared between WT and mutants by determining the area of the ALPL-positive domain within 50 μm from the edge of the frontal or parietal bone. Cell counts and area measurements were performed in Adobe Photoshop. TUNEL staining was performed using the In Situ Cell Death Detection Kit, Fluorescein (Roche, 11684795910) as described by the manufacturer. Images of histological sections were taken using a Nikon Eclipse E600 microscope equipped with a Nikon DS-Ri2 digital camera and NIS Elements (F4.30.01) software.

### Single-molecule fluorescent RNA in situ hybridization (smFISH)

smFISH was performed using the RNAscope Fluorescent Multiplex Reagent Kit (Advanced Cell Diagnostics, 320850) with modifications as described previously^13^. Probes (Advanced Cell Diagnostics) were for *Cd34* (319161-C3), *Clec3b* (539561-C2), *Col6a3* (552541-C2), *Col8a1* (518071), *Cxcl12* (422711-C3), *Erg* (546491), *Fxyd5* (527721-C2*)*, *Gli1* (311001-C2), *Hhip* (448441-C3), *Ibsp* (415501), *Igfbp3* (405941*), Mest* (405961), *Mmp13* (427601-C3), *Postn* (418581-C2), *Ptch1* (402811-C2), *Sox6* (472061-C2), *Spp1* (435191-C3), and *Top2a* (491221). Images were acquired on an AxioImager Z2M equipped with a 20x/0.8NA Zeiss Plan-Apochromat objective, a monochrome Axiocam 503 camera (Zeiss, 1936 × 1460 pixels, 4.54 µm × 4.54 µm per pixel, sensitivity ~400 nm–1000 nm) and Zen 2 Blue Edition software (version 2.0). Z-stack images were acquired at optimal sampling rate meeting Nyquist frequency requirements, as calculated by the software. Pseudo-colored images were made by converting grayscale images using the Color/Merge Channels function of Fiji (ImageJ, v2.1.0/1.53c).

### Microcomputed tomography (microCT)

The upper torso and head of embryos at E18.5 were fixed in 4% PFA for 48 h, equilibrated in PBS, and stored in PBS/0.1% sodium azide before microCT analysis at the Pennsylvania State University. Immediately prior to scanning, samples were embedded in a 50:50 mix of polyester and paraffin waxes (Electron Microscopy Sciences, 19312 and 19302-01, respectively) to prevent motion artifacts and desiccation. MicroCT images were acquired by the Center for Quantitative Imaging at the Pennsylvania State University on the General Electric v|tom|x L300 nano/microCT system using the 300-kV tube at 80 kV and 180 uA using a 0.2 mm aluminum filter and image voxel size of 0.015 mm isotropic. Image data were reconstructed on a 2024 × 2024-pixel grid as a 32-bit volume and were reduced to 16-bit. A minimum threshold of 70–100 mg/cm^3^ partial density hydroxyapatite (HA) based on HA phantoms imaged with the specimens was used to reconstruct three-dimensional (3D) isosurfaces of skulls for image analysis using Avizo 2019.3 (Thermo Fisher Scientific).

### Statistical evaluation of shape differences

3D coordinates of 39 biologically relevant landmarks (Supplementary Table 2) were collected from the isosurfaces. Landmark data were collected twice, data were checked for obvious measurement errors, and after correction, measurement error was minimized by averaging the coordinates of the two trials. Maximum accepted error in landmark placement was 0.05 mm. Variation in global skull shape was assessed by principal components analysis (PCA) using SAS 9.4 (SAS Institute). Variation in skull shape was assessed using PCA of 741 unique inter-landmark distances estimated from 39 landmarks representing the global shape of each skull. Inter-landmark distances were *ln*-transformed, and their variance-covariance matrix was used as the basis for the PCA following previously described methods^75^. The captured variation was projected onto a lower-dimensional space defined by principal components axes that are mutually orthogonal, linear combinations of the measurement data. The scores of an observation along the principal axes map that observation into the lower-dimensional space. Two types of PCA were performed: a PCA based on variation in form (size and shape together), followed by a PCA based on shape variation alone.

### Statistical analysis

Sample sizes (*n*) are given in the text or figure legends, with measurements being taken from distinct samples. For quantification of EdU incorporation and cell number, statistical analysis and dot plotting were performed with Microsoft Excel for Mac, version 16.16.27. Dot plots show the mean and standard deviation. Statistical significance was determined using the two-tailed Student’s *t*-test. *P*-values below 0.05 were considered significant.

### Reporting summary

Further information on research design is available in the Nature Research Reporting Summary linked to this article.

## Supplementary information

Supplementary Information

Description of Additional Supplementary Files

Supplementary Data 1

Supplementary Data 2

Reporting Summary

## Data Availability

Data for single-cell and bulk RNA-seq libraries reported in this study are available in the Gene Expression Omnibus (GEO) database and as part of the Transcriptome Atlases of the Craniofacial Sutures FaceBase2 project in the FaceBase data repository (facebase.org). The GEO accession number is “GSE178899”. The FaceBase accession numbers are “FB00000970 [10.25550/3TYP]” (single-cell RNA-seq) and “FB00000903 [10.25550/TJC]”, “FB00000902 [10.25550/TJY]”, “FB00000805 [10.25550/VHE]”, “FB00001076 [10.25550/1-71HY]”, and “FB00000998 [10.25550/1-3X0M]” (bulk RNA-seq). The CellPhoneDB default database is available at https://www.cellphonedb.org/. All other relevant data supporting the key findings of this study are available within the article and its Supplementary Information files or from the corresponding author upon reasonable request. Source data are provided with this paper.

## Source data

Source Data
