# Supplementary material for: Single-cell analysis identifies a key role for Hhip in murine coronal suture development
Source: Nat Commun. 2021 Dec 8;12:7132. doi: 10.1038/s41467-021-27402-5 (PMC8655033; doi:10.1038/s41467-021-27402-5)
Supplement: Supplementary file 1 — Supplementary Information [file 41467_2021_27402_MOESM1_ESM.pdf]

Supplementary Information

Single-cell analysis identifies a key role for *Hhip* in murine coronal suture development

Greg Holmes et al.

Supplementary Figures and Legends

Supplementary Tables

Supplementary Datasets

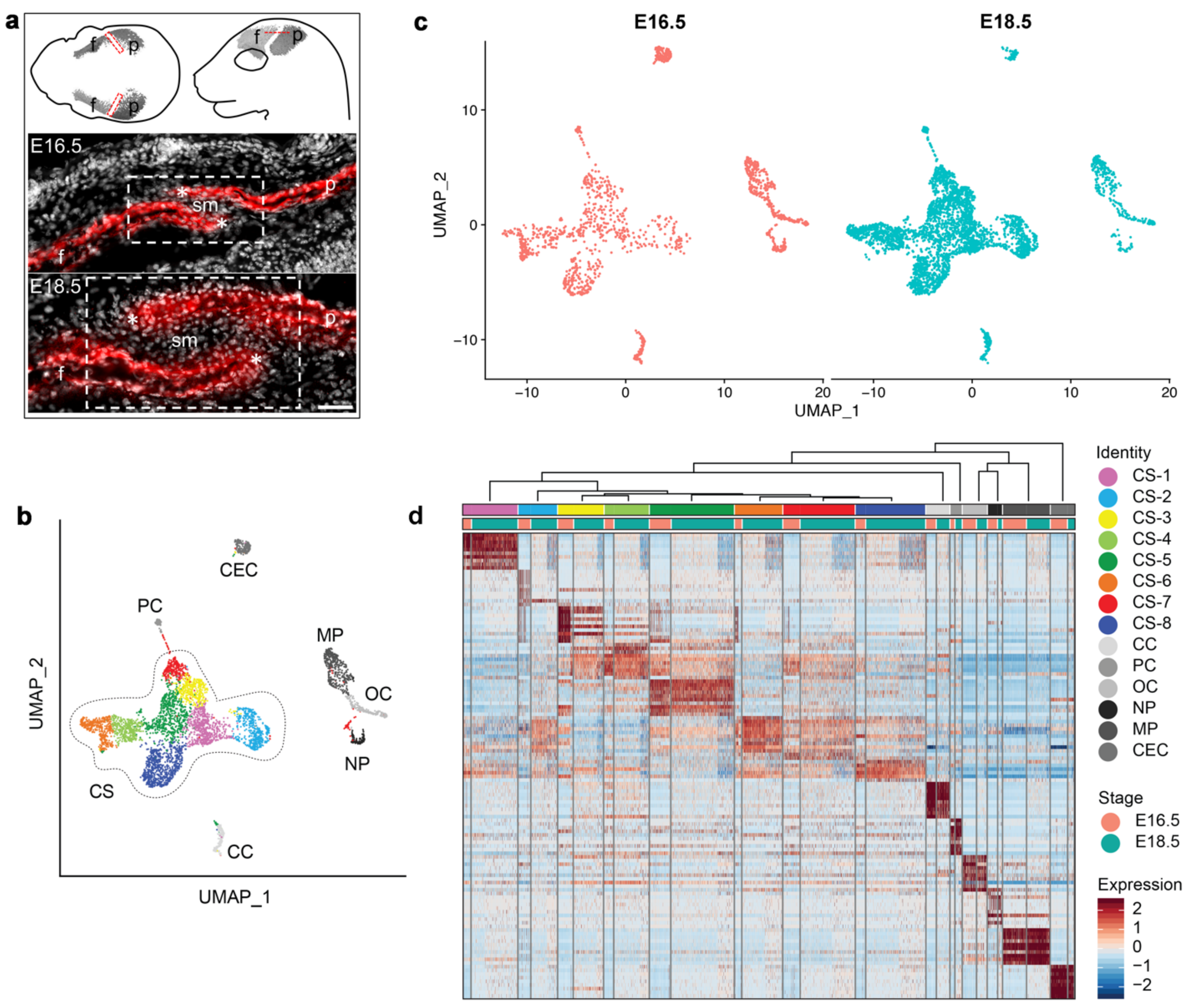

Supplementary Figure 1 Overview of coronal suture and combined analysis of single-cell populations at E16.5 and E18.5. a Isolation of coronal suture tissue for scRNA-seq analysis. At the top left, the schematic shows a top view of the E16.5 mouse head with the mineralized regions of the frontal (f) and parietal (p) bones represented by microcomputed tomography (microCT) images. At the top right, the schematic shows a side view of the E16.5 head. The lower two panels show transverse histological sections of the coronal suture at E16.5 and E18.5, stained for alkaline phosphatase activity (red) and DAPI (gray). Red dashed rectangles, regions between mineralized bone dissected

for single-cell analysis; red dashed line, transverse plane of sectioning for histological sections; white dashed rectangles, region of bone overlap included in suture dissections; white asterisks, osteogenic fronts; sm, suture mesenchyme. Scale bar, 50 µm. **b** Uniform Manifold Approximation and Projection (UMAP) plot of cell-type clusters detected by unsupervised graph clustering of cells from all replicates at E16.5 and E18.5. CS, coronal suture; CC, chondrocytes; CEC, capillary endothelial cells; MP, macrophages; NP, neutrophils; OC, osteoclasts; PC, pericytes. The supercluster of suture-specific cell populations (CS) is demarcated by a dashed line. Color code key is given in **d**. **c** Same as **b**, but with cells colored by developmental stage. **d** Heatmap of normalized expression of the top ten most significant marker genes for each identified cell population at E16.5 and E18.5 (FDR ≤ 0.05, lnFC ≥ 0.25). Columns represent individual cells and rows represent genes with increased (red) or decreased (blue) expression compared to the average (white) (see Supplementary Dataset 1).

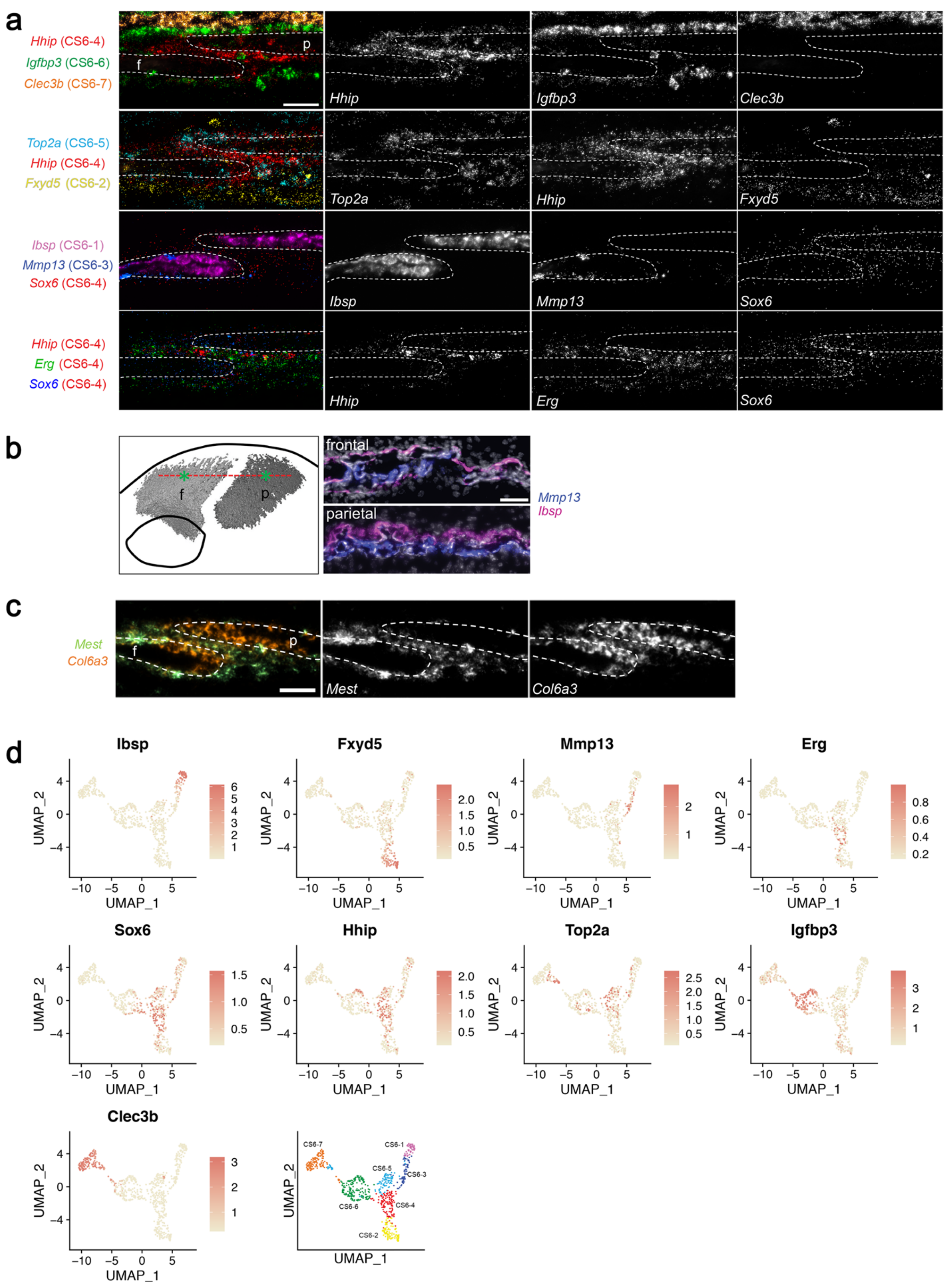

a
Hhip (CS6-4)
Igfbp3 (CS6-6)
Clec3b (CS6-7)
f
p
Hhip
Igfbp3
Clec3b
Top2a (CS6-5)
Hhip (CS6-4)
Fxyd5 (CS6-2)
Top2a
Hhip
Fxyd5
Ibsp (CS6-1)
Mmp13 (CS6-3)
Sox6 (CS6-4)
Ibsp
Mmp13
Sox6
Hhip (CS6-4)
Erg (CS6-4)
Sox6 (CS6-4)
Hhip
Erg
Sox6
b
f
p
frontal
parietal
Mmp13
Ibsp
c
Mest
Col6a3
f
p
Mest
Col6a3
d
Ibsp
Fxyd5
Mmp13
Erg
Sox6
Hhip
Top2a
Igfbp3
Clec3b
UMAP_1
UMAP_2
CS6-7
CS6-1
CS6-5
CS6-3
CS6-6
CS6-4
CS6-2

Supplementary Figure 2 Population expression mapping at E16.5. a Pseudo-colored images with indicated marker genes shown in Fig. 1c are reproduced at left. At bottom left, an additional panel showing markers for SM population CS6-4 is included. Grayscale images of smFISH expression for each marker gene are shown at right. f, frontal bone; p, parietal bone. Scale bar, 50 μm. **b** At left, the schematic shows the approximate level and position of transverse sections at right (red dashed line and green asterisks, respectively). Pseudo-colored images show *Mmp13* (blue) and *Ibsp* (magenta) expression in more central regions of the frontal and parietal bones at E16.5. DAPI-stained nuclei are shown in gray. The ectocranial and endocranial surfaces are at the top and bottom of each section, respectively. Scale bar, 50 μm. **c** Pseudo-colored image of *Mest* and *Col6a3* expression. Grayscale images of smFISH expression for each marker gene are shown at right. Scale bar, 50 μm. **d** UMAP plots showing expression of mapped, population-specific marker genes at E16.5 in order of CS6-1 to CS6-7. The color-coded UMAP plot of suture-specific populations (Fig. 1a) is included for reference. smFISH in **a-c** was performed on three independent samples with similar results.

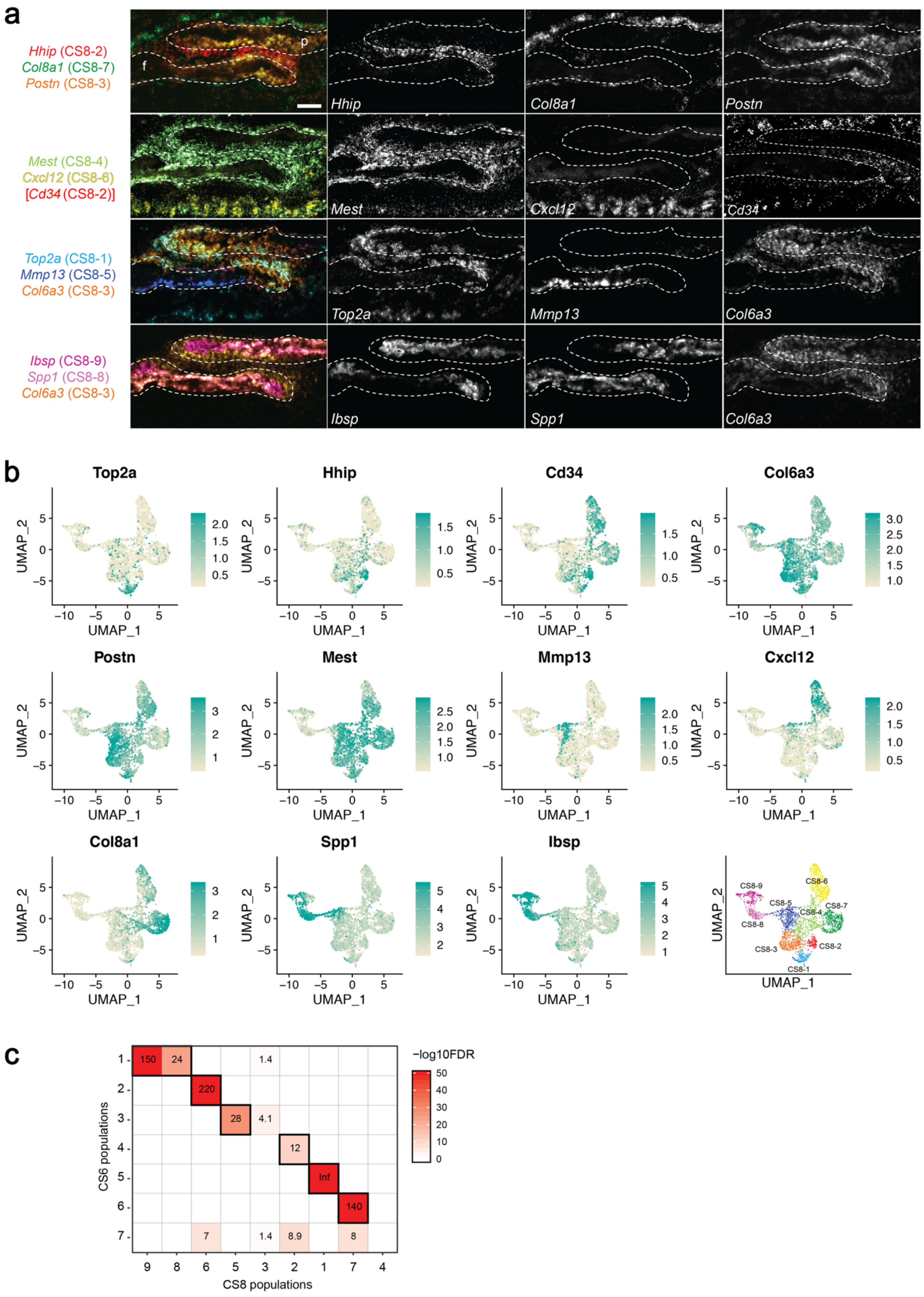
a
Hhip (CS8-2)
Col8a1 (CS8-7)
Postn (CS8-3)
p
f
Hhip
Col8a1
Postn
Mest (CS8-4)
Cxcl12 (CS8-6)
[Cd34 (CS8-2)]
Mest
Cxcl12
Cd34
Top2a (CS8-1)
Mmp13 (CS8-5)
Col6a3 (CS8-3)
Top2a
Mmp13
Col6a3
Ibsp (CS8-9)
Spp1 (CS8-8)
Col6a3 (CS8-3)
Ibsp
Spp1
Col6a3
b
Top2a
Hhip
Cd34
Col6a3
Postn
Mest
Mmp13
Cxcl12
Col8a1
Spp1
Ibsp
UMAP_1
UMAP_2
CS8-1
CS8-2
CS8-3
CS8-4
CS8-5
CS8-6
CS8-7
CS8-8
CS8-9
c
−log10FDR
150
24
1.4
220
28
4.1
12
Inf
140
7
1.4
8.9
8
CS6 populations
CS8 populations

Supplementary Figure 3 Population expression mapping at E18.5. a Pseudo-colored images with indicated marker genes shown in Fig. 2c are reproduced at left. Grayscale images of smFISH expression for each marker gene are shown at right. *Cd34*, an additional marker of CS8-2, is included in the second row. f, frontal bone; p, parietal bone. smFISH was performed on three independent samples with similar results. Scale bar, 50 µm. **b** UMAP plots showing expression of mapped, population-specific marker genes at E18.5 in order of CS8-1 to CS8-9. The color-coded UMAP plot of suture-specific populations (Fig. 2a) is included for reference. **c** Enrichment of gene expression signatures of the CS populations identified at E16.5 (*y*-axis, CS6) in those identified at E18.5 (*x*-axis, CS8). The -log10(*p*-value) is shown for the significantly enriched populations (FDR ≤ 0.05).

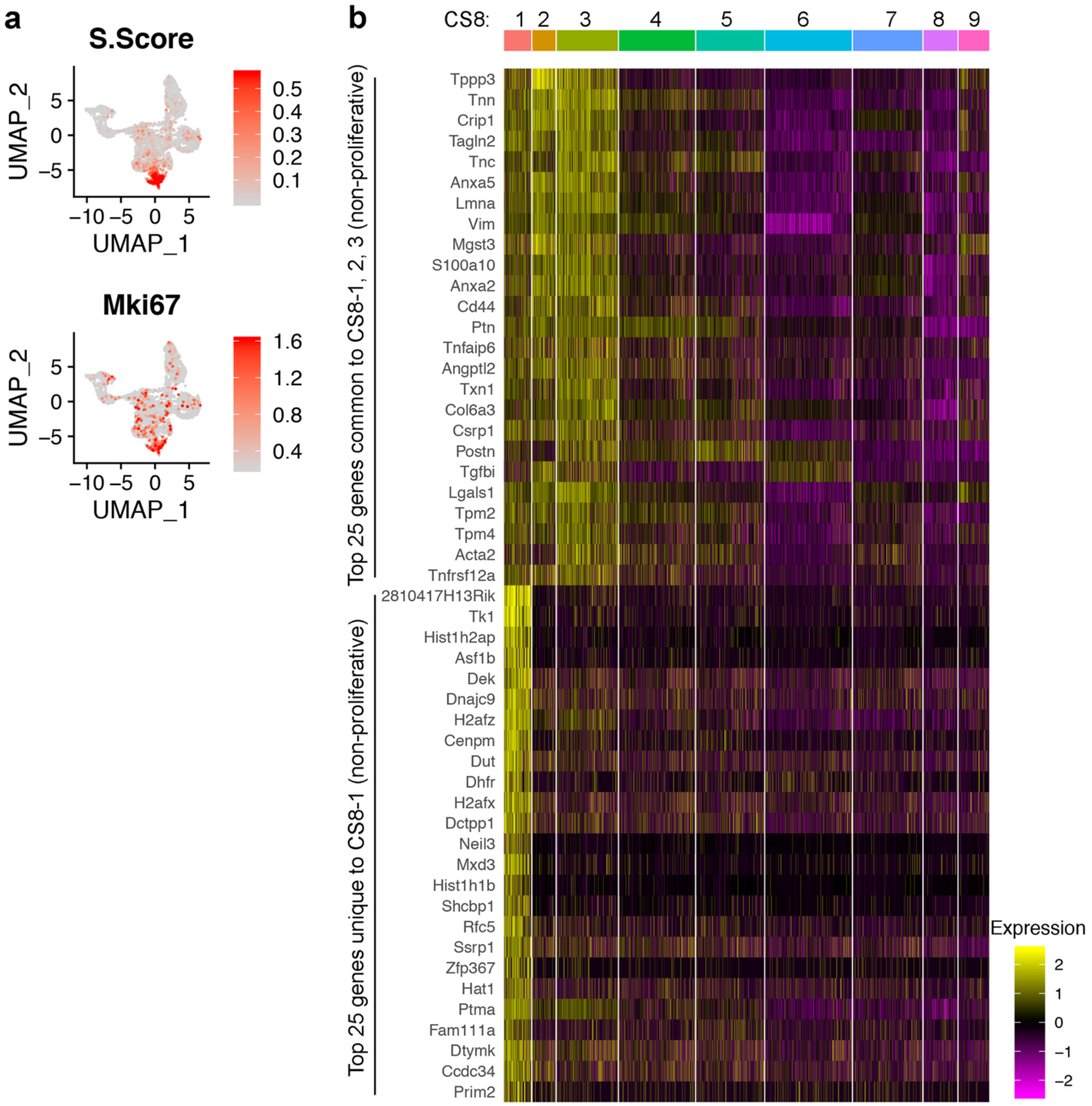

Supplementary Figure 4 Relationship of CS8-1, 2, and 3. a Distribution of proliferation (S) scores (top) and Mki67 expression (bottom) projected on a UMAP plot. **b** Heatmap of normalized expression of the top 25 most significant marker genes common to populations CS8-1, 2, and 3, and of the top 25 most significant marker genes exclusive to CS8-1, after removal of proliferation marker genes at E18.5 (FDR ≤ 0.05, lnFC ≥ 0.25).

Columns represent individual cells and rows represent genes with increased (yellow) or decreased (purple) expression compared to the average (black).

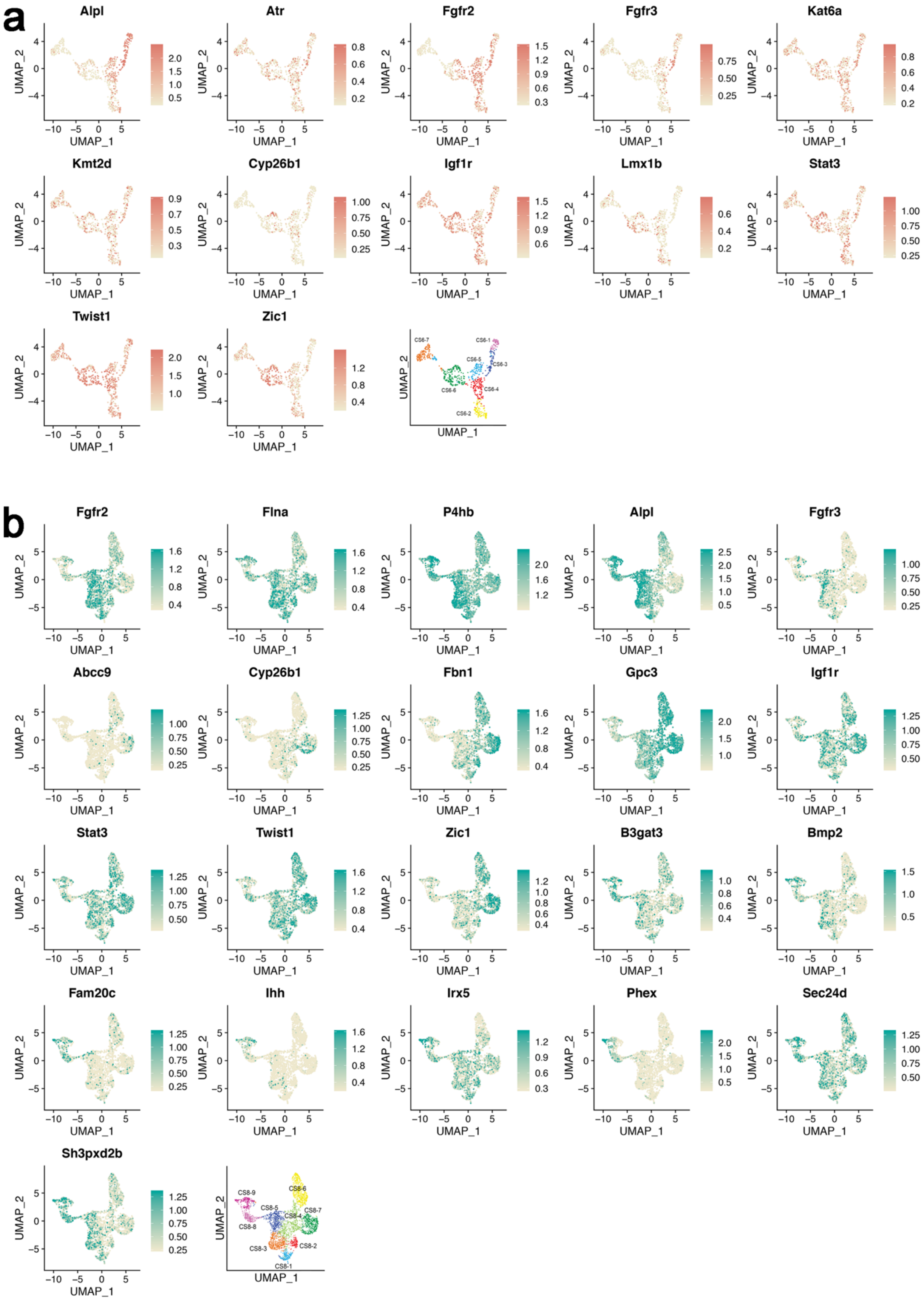

a
Alpl
Atr
Fgfr2
Fgfr3
Kat6a
Kmt2d
Cyp26b1
Igf1r
Lmx1b
Stat3
Twist1
Zic1
UMAP_1
UMAP_2
CS6-1
CS6-2
CS6-3
CS6-4
CS6-5
CS6-6
CS6-7
b
Fgfr2
Flna
P4hb
Alpl
Fgfr3
Abcc9
Cyp26b1
Fbn1
Gpc3
Igf1r
Stat3
Twist1
Zic1
B3gat3
Bmp2
Fam20c
Ihh
Irx5
Phex
Sec24d
Sh3pxd2b
CS8-1
CS8-2
CS8-3
CS8-4
CS8-5
CS8-6
CS8-7
CS8-8
CS8-9

Supplementary Figure 5 UMAPs of human craniosynostosis genes. a Expression distribution of craniosynostosis genes in specific coronal suture populations at E16.5 projected on a UMAP plot. The color-coded UMAP plot of suture-specific populations (Fig. 1a) is included for reference. **b** Expression distribution of craniosynostosis genes in specific coronal suture populations at E18.5 projected on a UMAP plot. The color-coded UMAP plot of suture-specific populations (Fig. 2a) is included for reference.

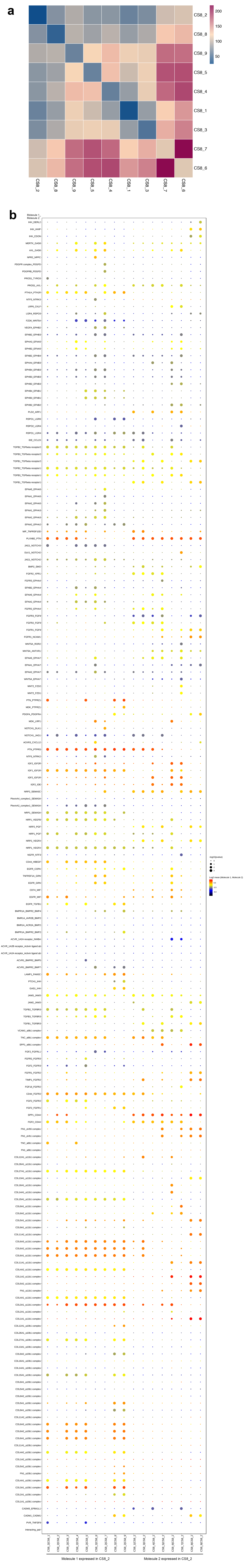
a
b
CS8_2
CS8_8
CS8_9
CS8_5
CS8_4
CS8_1
CS8_3
CS8_7
CS8_6
200
150
100
50
Molecule 1 expressed in CS8_2
Molecule 2 expressed in CS8_2

Supplementary Figure 6 Ligand/receptor analysis of E18.5 single-cell populations. **a** Heatmap showing the total number of ligand/receptor interactions (key at right) between cell types identified with CellPhoneDB. **b** Ligand/receptor interactions for genes expressed in CS8-2 (rows) between CS8-2 and the indicated populations (columns).

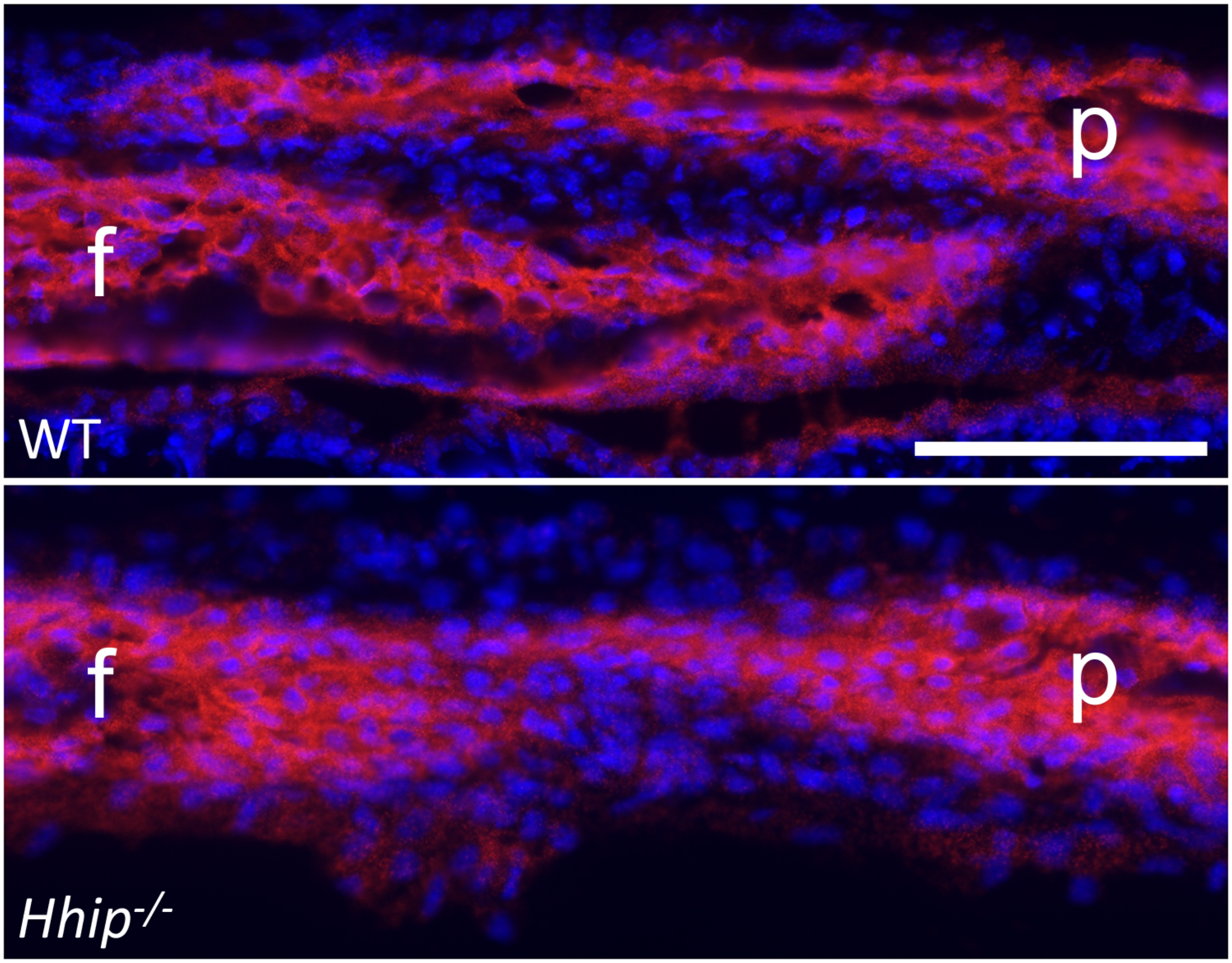

Supplementary Figure 7 *Hhip*$^{-/-}$ phenotype at P0. Staining for ALPL (red) in preosteoblasts and osteoblasts and for nuclei (DAPI, blue) for wild type (WT) and *Hhip*$^{-/-}$ coronal sutures. f, frontal bone; p, parietal bone. Results are representative of *n* = 3 WT and 3 *Hhip*$^{-/-}$ independent samples. Scale bar, 100 µm.

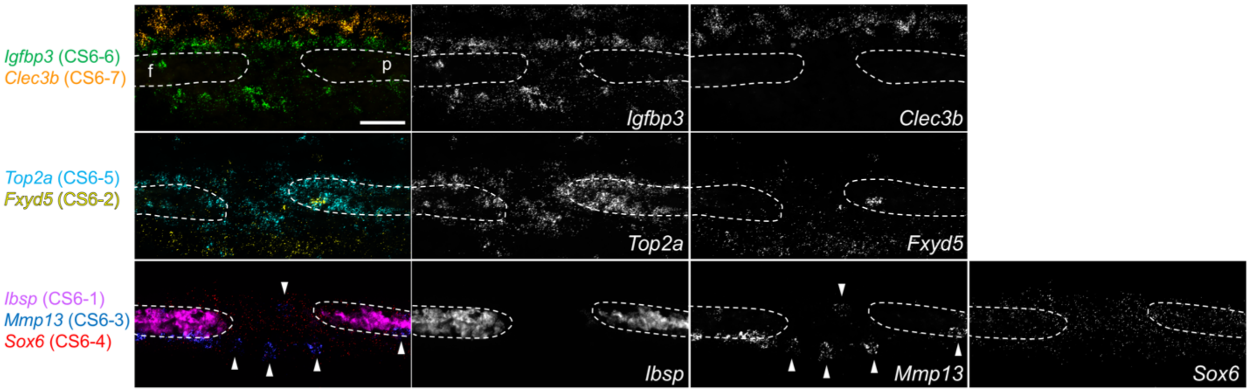

Supplementary Figure 8 Localization of E16.5 *Hhip*$^{-/-}$ coronal suture populations by smFISH. Pseudo-colored images with indicated marker genes shown in Fig. 8 are reproduced at left. Grayscale images of smFISH expression for each marker gene are shown at right. White arrowheads indicate novel *Mmp13* expression. Dashed outlines indicate frontal (f) and parietal (p) bones. Sections are in the transverse plane. smFISH was performed on three independent samples with similar results. Scale bar, 50 μm.

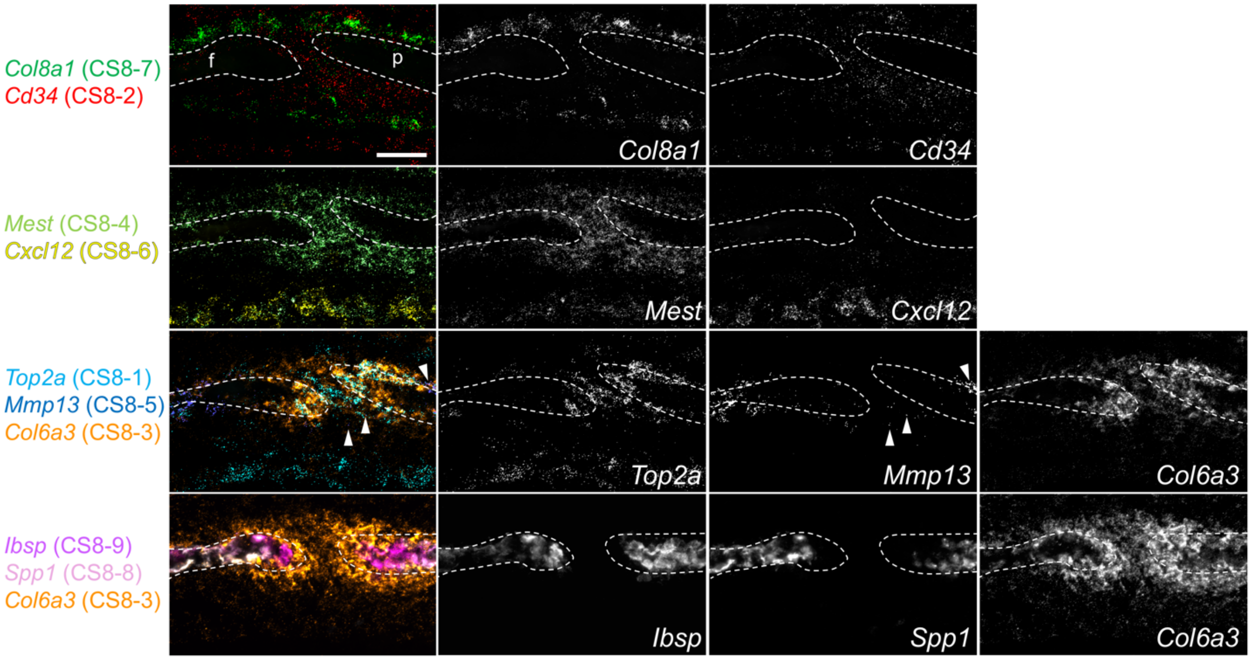

Supplementary Figure 9 Localization of E18.5 *Hhip*$^{-/-}$ coronal suture populations by smFISH. Pseudo-colored images with indicated marker genes shown in Fig. 8 are reproduced at left. Expression of the E18.5 CS8-2 marker, *Cd34*, can be compared to WT in Supplementary Fig. 3a. Grayscale images of smFISH expression for each marker gene are shown at right. White arrowheads indicate novel *Mmp13* expression. Dashed outlines indicate frontal (f) and parietal (p) bones. Sections are in the transverse plane. smFISH was performed on three independent samples with similar results. Scale bar, 50 μm.

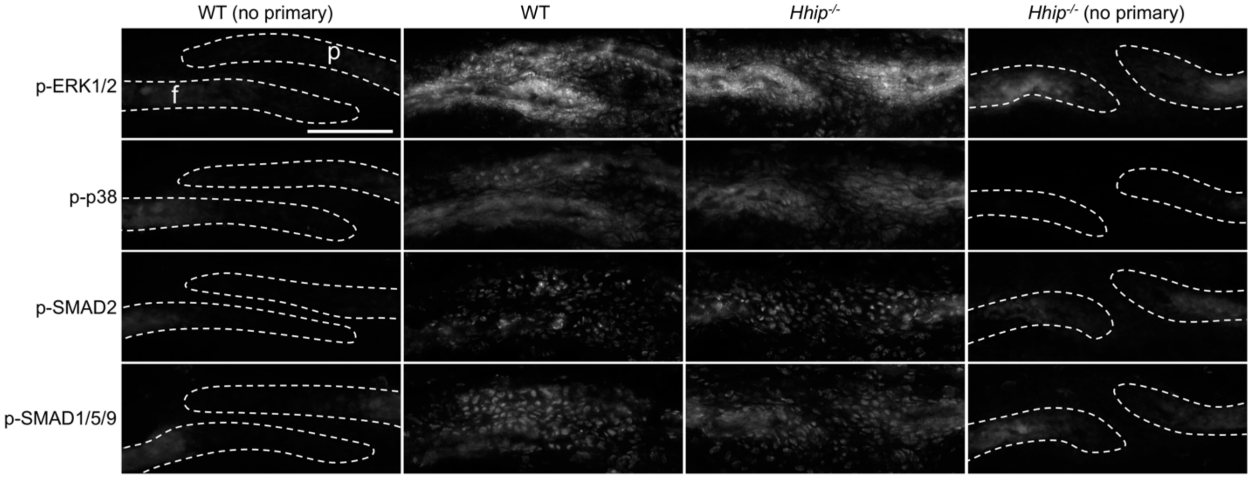

Supplementary Figure 10 FGF, TGF, and BMP signaling in the E18.5 *Hhip*$^{-/-}$ coronal suture. Immunohistochemistry for phospho (p)-ERK1/2 and p-p38 (FGF signaling), p-SMAD2 (TGF signaling), and p-SMAD1/5/9 (BMP signaling). Images are shown in grayscale. The first and last columns show background staining without primary antibody (no primary) from a section on the same slide as those in the second and third columns, respectively. Dashed outlines indicate frontal (f) and parietal (p) bones. Sections are in the transverse plane. Results presented are representative of *n* = 3 WT and 3 *Hhip*$^{-/-}$ independent samples. Scale bar, 100 μm.

Supplementary Table 1. Summary of E16.5 and E18.5 coronal suture populations.

Population	E16.5	E18.5
suture mesenchyme	CS6-4	CS8-2
peripheral suture mesenchyme	-	CS8-4
ectocranial suture mesenchyme	CS6-6	CS8-7
proliferating preosteoblast	CS6-5	CS8-1
early osteoblast	CS6-1	CS8-9
periosteal osteoblast	-	CS8-3
mature osteoblast	-	CS8-8
Mmp13-expressing osteoblast	CS6-3	CS8-5
hypodermis	CS6-7	not isolated
dura mater	CS6-2	CS8-6
chondrocyte	CC	CC
capillary endothelial cell	CEC	CEC
macrophage	MP	MP
neutrophil	NP	NP
osteoclast	OC	OC
pericyte	PC	PC

Supplementary Table 2. Anatomical definitions of skull landmarks used for PCA.

**Bilateral Landmarks**	**Anatomical Definition**
lnasapl, rnasapl	Most supero-anterior point of the premaxilla accounting for the lateral part of the nasal aperture
lfppm, rfppm	Most supero-posterior point of the premaxilla accounting for the lateral part of the nasal aperture
lnslp, rnslp	Most postero-medial point of the nasal bone
lnsla, rnsla	Most antero-medial point of the left nasal bone
liohd, riohd	Most distal point of the infraorbital hiatus
lflac, rflac	Intersection of frontal process of maxilla with frontal and lacrimal bones, taken on the maxilla
lzyt, rzyt	Intersection of zygoma with zygomatic process of temporal, taken on zygoma
lpfl, rpfl	Most lateral intersection of the frontal and parietal bones, taken on the parietal
lsqu, rsqu	Most superior point on the squamous temporal, intersection of the coronal suture
lpsq, rpsq	Most posterior point on the posterior extension of the forming squamosal
lpfm, rpfm	Most medial intersection of the frontal and parietal bones, taken on the parietal
lpto, rpto	Most postero-medial point on the parietal
loci, roci	The superior posterior point on the ectocranial surface of the occipital lateralis on the foramen magnum
lva, rva	Most posterior point on the ala of the vomer
lalf, ralf	Most anteromedial point on the frontal bone
lasph, rasph	Postero-medial point of the inferior portion of the alisphenoid
lsyn, rsyn	Most antero-lateral point on the corner of the basioccipital
**Individual Landmarks**	**Anatomical Definition**
ethma	Anterior most point on the body of the vomer, taken on the ventral surface
intpar	Most anterior point on the ectocranial surface of the interparietal on the midsagittal plane
ans	Anterior nasal spine is the most anterior point of interpremaxillary suture at base of nasal aperture, midline
amsph	Most antero-medial point on the body of the sphenoid
bas	Mid-point on the anterior margin of the foramen magnum, taken on basioccipital

Supplementary Dataset 1. scRNA-seq analysis of the coronal suture at E16.5 and E18.5.

Supplementary Dataset 2. Human and murine craniosynostosis genes.
