# Supplementary material for: Single-cell analysis identifies a key role for Hhip in murine coronal suture development
Source: Nat Commun. 2021 Dec 8;12:7132. doi: 10.1038/s41467-021-27402-5 (PMC8655033; doi:10.1038/s41467-021-27402-5)
Supplement: Supplementary file 2 — Description of Additional Supplementary Files [file 41467_2021_27402_MOESM2_ESM.pdf]

Description of Additional Supplementary Files

Title: Supplementary Data 1

Description: Coronal suture scRNA-seq analysis of cell type identification and gene ontology

Title: Supplementary Data 2

Description: Human and murine craniosynostosis genes
