# Supplementary material for: Single-cell analysis identifies a key role for Hhip in murine coronal suture development
Source: Nat Commun. 2021 Dec 8;12:7132. doi: 10.1038/s41467-021-27402-5 (PMC8655033; doi:10.1038/s41467-021-27402-5)
Supplement: Supplementary file 5 — Reporting Summary [file 41467_2021_27402_MOESM5_ESM.pdf]

nature research

Corresponding author(s): Greg Holmes

Last updated by author(s): Oct 28, 2021

Reporting Summary

Nature Research wishes to improve the reproducibility of the work that we publish. This form provides structure for consistency and transparency in reporting. For further information on Nature Research policies, see our Editorial Policies and the Editorial Policy Checklist.

Statistics

For all statistical analyses, confirm that the following items are present in the figure legend, table legend, main text, or Methods section.

n/a	Confirmed	
☐	☒	The exact sample size (*n*) for each experimental group/condition, given as a discrete number and unit of measurement
☐	☒	A statement on whether measurements were taken from distinct samples or whether the same sample was measured repeatedly
☐	☒	The statistical test(s) used AND whether they are one- or two-sided<br>*Only common tests should be described solely by name; describe more complex techniques in the Methods section.*
☐	☒	A description of all covariates tested
☐	☒	A description of any assumptions or corrections, such as tests of normality and adjustment for multiple comparisons
☐	☒	A full description of the statistical parameters including central tendency (e.g. means) or other basic estimates (e.g. regression coefficient) AND variation (e.g. standard deviation) or associated estimates of uncertainty (e.g. confidence intervals)
☐	☒	For null hypothesis testing, the test statistic (e.g. *F*, *t*, *r*) with confidence intervals, effect sizes, degrees of freedom and *P* value noted<br>*Give P values as exact values whenever suitable.*
☒	☐	For Bayesian analysis, information on the choice of priors and Markov chain Monte Carlo settings
☐	☒	For hierarchical and complex designs, identification of the appropriate level for tests and full reporting of outcomes
☒	☐	Estimates of effect sizes (e.g. Cohen's *d*, Pearson's *r*), indicating how they were calculated

Our web collection on statistics for biologists contains articles on many of the points above.

Software and code

Policy information about availability of computer code

Data collection	No software was used for data collection.
Data analysis	10X Genomics Cellranger v.3 was used to process raw sequencing data.<br>Seurat package v3.1.1 was used for scRNA-seq analysis in conjunction with mvoutlier R package v2.0.8 and gProfileR v0.6.4 .<br>Cell communication analysis was performed with CellphoneDB v2.0<br>Trajectory analysis with UMAP and approximate graph abstraction was performed with Monocle3.<br>For microCT analysis, reconstruction of three-dimensional (3D) isosurfaces of skulls for image analysis was performed using Avizo 2019.3 and variation in global skull shape was assessed by principal components analysis using SAS 9.4 (SAS Institute).

For manuscripts utilizing custom algorithms or software that are central to the research but not yet described in published literature, software must be made available to editors and reviewers. We strongly encourage code deposition in a community repository (e.g. GitHub). See the Nature Research guidelines for submitting code & software for further information.

Data

Policy information about availability of data

All manuscripts must include a data availability statement. This statement should provide the following information, where applicable:
- Accession codes, unique identifiers, or web links for publicly available datasets
- A list of figures that have associated raw data
- A description of any restrictions on data availability

Data for single-cell and bulk RNA-seq libraries reported in this study are available in the Gene Expression Omnibus (GEO) database and as part of the Transcriptome Atlases of the Craniofacial Sutures FaceBase2 project in the FaceBase data repository (facebase.org). The GEO accession number is GSE178899 (https://www.ncbi.nlm.nih.gov/geo/query/acc.cgi?acc=GSE178899). The FaceBase accession numbers are FB00000970 (https://doi.org/10.25550/3TYP) (single-cell RNA-

seq) and FB00000903 (https://doi.org/10.25550/TJC), FB00000902 (https://doi.org/10.25550/TJY), FB00000805 (https://doi.org/10.25550/VHE), FB00001076 (https://doi.org/10.25550/1-71HY), and FB00000998 (https://doi.org/10.25550/1-3X0M) (bulk RNA-seq). The CellPhoneDB default database is available at https://www.cellphonedb.org/. Source data are provided with this paper.

Field-specific reporting

Please select the one below that is the best fit for your research. If you are not sure, read the appropriate sections before making your selection.

☒ Life sciences ☐ Behavioural & social sciences ☐ Ecological, evolutionary & environmental sciences

For a reference copy of the document with all sections, see nature.com/documents/nr-reporting-summary-flat.pdf

Life sciences study design

All studies must disclose on these points even when the disclosure is negative.

Sample size	For single-cell experiments two replicates were performed at each age, as considered sufficient in the field. For wild type versus Hhip-/- comparisons, a minimum of 3 of each genotype were used, as considered standard in the field.
Data exclusions	No data was excluded from the analysis.
Replication	For scRNA-seq experiments two independent libraries at each age were generated. Qualitative validation of RNA expression patterns in histological sections was performed in a minimum of 3 samples of the indicated genotypes. For quantification in histological sections the sample size of n=3 or 6 each for wild type and Hhip-/- genotypes is given in the text or figure legends and included mice from different litters. For microCT experiments a minimum of 4 samples of each genotype were used, as indicated in the figure legend. All qualitative findings could be replicated. The significance of quantitative differences between the indicated n for each genotype are as reported in the figure legends.
Randomization	For single-cell RNA-seq experiments only wild type mice were used and all mice in a litter were used at the time of library preparation. For in situ hybridization/immunohistochemical assays and microCT assays the reported experiments did not consist of treatment/non-treatment groups. Rather, samples (mice) were distinguished by genotype (wild type versus Hhip-/-) and assessed for the same experimental measure (e.g., cell proliferation, immunohistochemical staining, distance between landmarks).
Blinding	For single-cell RNA-seq experiments only wild type mice were used and blinding was not an issue. Due to the obvious phenotype differences between wild type and Hhip-/- samples, blinding during data collection was not feasible for in situ hybridization/immunohistochemical assays. For microCT analysis, the researcher was blinded to wild type and Hhip-/- genotypes.

Reporting for specific materials, systems and methods

We require information from authors about some types of materials, experimental systems and methods used in many studies. Here, indicate whether each material, system or method listed is relevant to your study. If you are not sure if a list item applies to your research, read the appropriate section before selecting a response.

Materials & experimental systems

n/a	Involved in the study
☐	☒ Antibodies
☒	☐ Eukaryotic cell lines
☒	☐ Palaeontology and archaeology
☐	☒ Animals and other organisms
☒	☐ Human research participants
☒	☐ Clinical data
☒	☐ Dual use research of concern

Methods

n/a	Involved in the study
☒	☐ ChIP-seq
☒	☐ Flow cytometry
☒	☐ MRI-based neuroimaging

Antibodies

Antibodies used	anti-RUNX2, Sigma-Aldrich, Cat# HPA022040, Lot# B97117; anti-SP7/Osterix, Abcam, Cat# ab22552, Lot # GR3357012-1; anti-phospho-P42/44 MAPK (ERK1/2), Cell Signaling Technology, Cat# 4376, Lot# 17; anti-phospho-p38 MAPK, Cell Signaling Technology, Cat# 4631), Lot# 7; anti-phospho-SMAD2, Cell Signaling Technology, Cat# 3108, Lot# 10; and anti-phospho-SMAD1/5/8(9), Cell Signaling Technology, Cat# 9511, Lot# 11.
Validation	Antibodies used are commercially available.<br><br>The anti-RUNX2 antibody is validated by Sigma for immunoblotting, immunofluorescence, and immunohistochemistry. As a Prestige Antibody it is tested in the following ways: IHC tissue array of 44 normal human tissues and 20 of the most common cancer type tissues, and protein array of 364 human recombinant protein fragments.<br><br>The anti-SP7 antibody is validated by Abcam for immunohistochemistry and reacts with mouse, rat, and human SP7.

The anti-pERK1/2 antibody is validated by Cell Signaling for immunohistochemistry and reacts with Human, Mouse, Rat, Hamster, Monkey, Mink, D. melanogaster, Zebrafish, Pig, S. cerevisiae. The antibody does not cross-react with the corresponding phosphorylated residues of either JNK/SAPK or p38 MAP kinase.

The anti-pP38 antibody is validated by Cell Signaling for immunohistochemistry and reacts with Human, Mouse, Rat, Monkey, D. melanogaster. This antibody does not cross-react with the phosphorylated forms of either p42/44 MAPK or SAPK/JNK.

The anti-pSMAD2 antibody is validated by Cell Signaling to react with Human, Mouse, Rat, and Mink. This antibody does not cross-react with other Smad-related proteins.

The anti-pSMAD1/5/8(9) antibody is validated by Cell Signaling for immunohistochemistry and reacts with Human, Mouse, Mink, and Xenopus. This antibody does not cross-react with other Smad-related proteins.

Animals and other organisms

Policy information about studies involving animals; ARRIVE guidelines recommended for reporting animal research

Laboratory animals	Mouse procedures were in compliance with animal welfare guidelines mandated by the Institutional Animal Care and Use Committee (IACUC) of the Icahn School of Medicine at Mount Sinai and the Pennsylvania State University. C57BL/6J mice (The Jackson Laboratory, 000664), Hhiptm1Amc/J mice (The Jackson Laboratory, 006241), Hhip-EGFP_T2A_CreERT2 mice (European Mouse Mutant Archive, 12335) and Ai14 mice (The Jackson Laboratory, 007914) were used in this study. Water and food were available ad libitum, and mice were maintained on a 12:12 hour light:dark cycle at a temperature of 20-22 degrees Celsius and 30-70% humidity. Data was collected from embryos at E16.5 and E18.5 or post-natal mice at various ages as specified in the manuscript. Embryos or postnatal mice of both sexes were included in all experiments. No sex difference is expected in the parameters studied at the reported ages.
Wild animals	Not applicable to this study.
Field-collected samples	Not applicable to this study.
Ethics oversight	Mouse procedures were in compliance with animal welfare guidelines mandated by the Institutional Animal Care and Use Committee (IACUC) of the Icahn School of Medicine at Mount Sinai and the Pennsylvania State University.

Note that full information on the approval of the study protocol must also be provided in the manuscript.
